# The Researchers’ View of Scientific Rigor—Survey on the Conduct and Reporting of *In Vivo* Research

**DOI:** 10.1371/journal.pone.0165999

**Published:** 2016-12-02

**Authors:** Thomas S. Reichlin, Lucile Vogt, Hanno Würbel

**Affiliations:** Division of Animal Welfare, Veterinary Public Health Institute, Vetsuisse Faculty, University of Bern, Bern, Switzerland; Van Andel Institute, UNITED STATES

## Abstract

Reproducibility in animal research is alarmingly low, and a lack of scientific rigor has been proposed as a major cause. Systematic reviews found low reporting rates of measures against risks of bias (e.g., randomization, blinding), and a correlation between low reporting rates and overstated treatment effects. Reporting rates of measures against bias are thus used as a proxy measure for scientific rigor, and reporting guidelines (e.g., ARRIVE) have become a major weapon in the fight against risks of bias in animal research. Surprisingly, animal scientists have never been asked about their use of measures against risks of bias and how they report these in publications. Whether poor reporting reflects poor use of such measures, and whether reporting guidelines may effectively reduce risks of bias has therefore remained elusive. To address these questions, we asked in vivo researchers about their use and reporting of measures against risks of bias and examined how self-reports relate to reporting rates obtained through systematic reviews. An online survey was sent out to all registered in vivo researchers in Switzerland (N = 1891) and was complemented by personal interviews with five representative in vivo researchers to facilitate interpretation of the survey results. Return rate was 28% (N = 530), of which 302 participants (16%) returned fully completed questionnaires that were used for further analysis. According to the researchers’ self-report, they use measures against risks of bias to a much greater extent than suggested by reporting rates obtained through systematic reviews. However, the researchers’ self-reports are likely biased to some extent. Thus, although they claimed to be reporting measures against risks of bias at much lower rates than they claimed to be using these measures, the self-reported reporting rates were considerably higher than reporting rates found by systematic reviews. Furthermore, participants performed rather poorly when asked to choose effective over ineffective measures against six different biases. Our results further indicate that knowledge of the ARRIVE guidelines had a positive effect on scientific rigor. However, the ARRIVE guidelines were known by less than half of the participants (43.7%); and among those whose latest paper was published in a journal that had endorsed the ARRIVE guidelines, more than half (51%) had never heard of these guidelines. Our results suggest that whereas reporting rates may underestimate the true use of measures against risks of bias, self-reports may overestimate it. To a large extent, this discrepancy can be explained by the researchers’ ignorance and lack of knowledge of risks of bias and measures to prevent them. Our analysis thus adds significant new evidence to the assessment of research integrity in animal research. Our findings further question the confidence that the authorities have in scientific rigor, which is taken for granted in the harm-benefit analyses on which approval of animal experiments is based. Furthermore, they suggest that better education on scientific integrity and good research practice is needed. However, they also question reliance on reporting rates as indicators of scientific rigor and highlight a need for more reliable predictors.

## Introduction

Reproducibility is the cornerstone of the scientific method and fundamental for the ethical justification of in vivo research. Mounting evidence of poor reproducibility (e.g. [1,2]) and translational failure of preclinical animal research [3–5] has therefore raised serious concerns about the scientific validity [6,7] and ethical justification [8,9] of in vivo research. Possible reasons for poor reproducibility include a lack of education [10,11], perverse incentives [12], ignorance of standards of good research practice [2], as well as scientific misconduct and fraud [13]. All of these may result in poor experimental design and conduct, thereby compromising scientific validity [5,14–17].

Poor scientific validity has important scientific, economic, and ethical implications. It hampers scientific and medical progress and leads to translational failure through misguided research efforts (e.g. [3,18–21]). It also increases R&D costs in drug development [22], resulting in higher health care costs (e.g. [17]). Based on estimates of irreproducibility in preclinical research, up to USD 28B/year may be spent in the US alone on irreproducible preclinical research [19]. Furthermore, poor scientific validity imposes unnecessary harm and distress upon research animals (e.g. [8,9]), raises false hopes in patients awaiting cures for their diseases, and puts patients in clinical trials at risk [23].

Much of the evidence of poor experimental design and conduct in animal research rests on systematic reviews and meta-analyses revealing low rates of reporting of measures against risks of bias (e.g., randomization: mean = 27% [range = 9–55%], blinding: 28.7% [0–61%], sample size calculation: 0.5% [0–3%]) in the primary literature (e.g. [23,24–33]). Consequently, reporting guidelines such as the ‘Animal Research: Reporting of In Vivo Experiments’ (ARRIVE) guidelines [34] (https://www.nc3rs.org.uk/arrive-guidelines) or the revised ‘Reporting Checklist for Life Science Articles’ by the Nature publishing group (http://www.nature.com/authors/policies/reporting.pdf) were promoted in view of improving the situation. For example, the ARRIVE guidelines consist of a checklist of 20 items of information that all publications reporting animal research should include, including details of methods used to reduce bias such as randomisation and blinding. Despite general consensus about the benefits of such guidelines (> 1000 journals have endorsed the ARRIVE guidelines by September 2016), Baker et al. [35] found that reporting rates of measures against bias remained low in PLoS and Nature journals even after they had endorsed the ARRIVE guidelines. Although reporting rates are generally increasing, they are still rather low [26].

In the past, the reporting of measures against bias such as randomization, blinding, sample size calculation and others was largely optional and–to some extent–this is still the case today. Therefore, reporting rates of these measures may not be reliable indicators of scientific rigor. Whether poor reporting reflects poor scientific validity, however, has never been systematically studied. Nevertheless, some indications exist that scientific rigor is often lacking, and that risks of bias are associated with poor reporting. For example, in neuroscience research most experimental studies are underpowered, and low statistical power in combination with null hypothesis significance testing and publication bias may lead to inflated effect size estimates from the published literature (e.g. [36]); inappropriate statistical methods often lead to spurious conclusions (e.g. [37,38,39]); and several systematic reviews indicate that low reporting rates of measures against bias are associated with larger effect sizes (e.g. [28,29,30,40]). This has raised concerns that there may be systemic flaws in the way we conduct and report research [2]. Several authors warned that the quality of animal research is (unacceptably) poor (e.g. [41]) and stricter adherence to standards of best research practice is necessary if the scientific validity of animal research is to be improved [15].

In light of the many studies published on poor reporting of measures against bias and the level of attention they received [5,7,18,42], it is surprising that so far no study has investigated the relationship between what researchers do in the laboratory and what they report in their publications. The primary aim of the present study, therefore, was to assess the researchers’ view of the quality of experimental conduct and how this relates to what they report in the primary literature. Using a questionnaire sent out to all registered animal scientists actively involved with ongoing animal experiments in Switzerland, we assessed (i) the researchers’ awareness and knowledge of risks of bias in animal research, (ii) the measures they take to avoid bias in their own research, and (iii) how they report these measures in their publications. To aid interpretation of the results, we also conducted qualitative interviews with a small subset of these researchers to get insight into personal viewpoints, underlying motivations, and compliance with quality standards.

## Methods

### Online survey

An anonymous online survey was developed using the free software Limesurvey [43]. The survey contained a total of 21 questions divided into seven sections. Thus, participants were asked about (i) their area of research, and the species they were mainly working with; (ii) their work institution, including certification; (iii) experimental design and conduct, including which of seven primary measures against risks of bias (Table 1) participants generally apply to their own research; (iv) the journal of their latest scientific publication and which of the seven measures against bias listed in Table 1 they had reported in that publication (or the reasons for not reporting them); (v) awareness of risks of bias, and knowledge about measures to prevent them; (vi) familiarity with the ARRIVE (or similar) guidelines, and whether they adhered to them; and (vii) the participants’ personal research experience. The questionnaire was piloted among five animal researchers to ensure clarity. Participants had to answer all questions of a section before being able to move on to the next section, however, for most questions they had the option of not answering questions by ticking ‘no answer’, ‘do not know’, or ‘not relevant’. The full questionnaire is available in S1 Text.

**Table 1:** List of measures against risks of bias included in this study.

Measure	Definition	Bias
Allocation Concealment	Concealment of allocation sequence from those assigning subjects to treatment groups, until the moment of assignment.	Selection Bias
Randomization	Allocation of study subjects randomly to treatment groups across the comparison, to ensure that group assignment cannot be predicted.	Selection Bias
Blinding	Keeping the persons involved in an experiment (i.e. experimenter, data collector, outcome assessors) unaware of the treatment allocation.	Attrition Bias, Detection Bias, Performance Bias
Sample Size Calculation	Appropriate *a priori* determination of number of study subjects for a given test setup that allows for a detection of a treatment effect given the power to find an effect of a defined size.	“Avoiding wastage of animals”
Inclusion and Exclusion Criteria	*A priori* defined characteristics which describe on which basis subjects will be included in the study or how they need to be treated in case of attrition.	Attrition Bias, Selective Reporting
Primary Outcome	*A priori* defined main variable of interest, on which the treatment effect is measured; with sample size calculation being based on it.	Selective Reporting
Statistical Analysis Plan	*A priori* definition of statistical methods by which the primary outcome variable is analyzed at the end of the study.	Attrition Bias, Selective Reporting

Definitions adapted from van der Worp et al. [5], CONSORT (www.consort-statement.org), the Cochrane Collaboration (methods.cochrane.org).

### Study population and data collection

The online survey was set up as a partially closed survey, for which potential participants (N = 1891) were invited via email. Email addresses were provided by the Swiss Federal Food Safety and Veterinary Office (FSVO) and included all researchers involved in ongoing animal experiments in Switzerland, which were registered by the FSVO as experimenters, study directors, or resource managers of animal facilities. The questionnaire was online for seven weeks; after five weeks a reminder for participation was sent to all addressees to increase response rate.

### Ethics statement

Given that there were no known risks associated with this research study, participants of the survey and the interviews were not a vulnerable group of people, and complete confidentiality was guaranteed, we saw no need for formal ethical review before the study began.

### Data analysis

The online survey generated 530 questionnaires (return rate: 28%), of which 302 (57%) were fully completed while 228 were only partially completed. Partially completed questionnaires were only used for assessing a potential bias in the sample of fully completed questionnaires, while only the latter (16% of the total sample) were used for further analysis. Survey data were exported to MS Excel, checked for inconsistencies, and revised if necessary with suitable correction rules. Each question of the survey was analyzed quantitatively in terms of proportions of the answers given by the participants.

Besides analyzing each question separately, internal validity scores (IVS) were calculated for each participant for a) experimental conduct (IVS_Exp_), and b) reporting in the latest publication (IVS_Pub_). The scores were based on the measures against risks of bias (Table 1) and were equal to the number of these measures claimed to be applied (under a, Eq 1) or reported (under b, Eq 2) by the participant, divided by the number of measures that were applicable to a) or b), respectively.

$${IVS}_{Exp}=numberofyesanddepends/\left(7-numberofno\;answer\right)$$ (1)

IVSPub=number of yes (full details)-(6-Σ(numbers of no answer+does not apply to last manuscript+have not published yet)). (2)

For an overview of all possible answer options, please refer to the copy of the online survey in S1 Text. Due to a mistake in the way the questions regarding allocation concealment were formulated, data for this measure were excluded from both IVS in the case of a direct comparison between the scores (change of denominator in Eq 1 to “6—number of *no answer*”). In addition, the influence of several independent variables (descriptors of the participants derived from the online survey) on these scores was investigated through an information theoretic modelling approach using generalized linear models (*glm*). The Bayesian Information Criterion (BIC) was used to compare candidate models [44] and to retrieve the model which best described the data [45]. The two scores were modelled with the following main effects (descriptors): Knowledge of the ARRIVE guidelines (binary; yes, no), host institution (categorical; academia, industry, governmental, private), animal research experience (continuous; no. of years), the authority (cantonal veterinary office) responsible for approving the participants’ applications for animal experiments (categorical; 13 cantons), field of research (categorical: basic, applied, other), and the research discipline (categorical: Animal Welfare, Cell Biology/Biochemistry/Molecular Biology, Ethology, Human Medicine, Para-clinics, Veterinary Medicine, Zoology, other discipline).

Starting with the full model (all descriptors including the interaction term knowledge of ARRIVE x institution), single term deletion was performed by a stepwise backwards procedure (*drop1* function), eliminating the descriptor with the largest p-value to produce a set of candidate models for the model selection process. Besides this set of candidate models, we also included all univariate models (single descriptors) as well as the null model (only intercept; total of 12 models). The model comparison was conducted using the function *model*.*sel* from the R-package MuMIn [46]. The model with the lowest BIC was chosen as the one fitting the data best. Model estimates and 95% confidence intervals were corrected for overdispersion of the data (*glm* link function = quasibinomial).

In order to investigate whether the IVS_Exp_ and IVS_Pub_ were correlated, a Spearman’s Rank Correlation was performed with the reduced IVS scores (only considering six validity criteria, i.e., without allocation concealment). Mean differences in IVS between participants of certified institutions vs. non-certified institutions were investigated with a Wilcoxon Rank Sum Test for both scores. Values for IVS are presented as means ± SD. All statistical analysis were performed using the statistical software R, Version 3.0.3 [47].

### Personal Interviews

The online survey was complemented by interviews with five selected researchers representing the diversity of institutions and areas of research among the participants of the survey. The interviews are not described in the main text of this article, however, complete information about the methods, study population, analysis and results of the interviews is provided in S2 Text.

## Results

### Study population

The 302 participants returning fully completed questionnaires had an average of 15.5 (SD ± 8.6) years of experience in animal research. Most of them were affiliated with academic institutions (74.5%, N = 225), 14.9% (N = 45) with industry, 4.6% (N = 14) with governmental research institutions, and 6% (N = 18) with private research institutions. Only 23.8% of the participants (N = 72) indicated that their institution was formally certified, and 27.2% (N = 82) that it was not, while almost half of the participants (44.7%) did not know this or did not answer this question (4.3%) (for a complete table, see S1 Table). Among the 72 participants indicating that their institution was certified, academics were relatively underrepresented with only 45.8% compared to 74.5% among the total sample, whereas researchers from pharmaceutical industry (29.2% vs. 14.9%), governmental institutions (8.3% vs. 4.6%), and private institutions (16.7% vs. 6%) were relatively overrepresented.

Most participants (58.9%, N = 178) attributed their work to basic research and 40.4% (N = 122) to applied research, while two participants (0.7%) were undecided. The large majority of participants (86.8%) were engaged in biomedical or medical research (for details see Table 2), and the animals used as experimental subjects were mainly mice (60.6%) and rats (15.6%) (for details see Table 3). While 14 participants (4.6%) had not yet published their first paper, most participants (57%, N = 172) had published between 1 and 20 papers, and 116 participants (38.4%) had published more than 20 papers.

**Table 2:** Research Disciplines of Survey Participants.

Discipline	Number	Proportion [%]
Human Medicine	108	36
Biochemistry, Cell or Molecular Biology	105	35
Veterinary Medicine	27	9
Para-clinics	22	7
Other	15	5
Animal Welfare	12	4
Zoology	11	4
Ethology	2	1

**Table 3:** Primary Research Species of Survey Participants.

Primary Species	Number	Proportion [%]
Mice	183	60.60
Rats	47	15.56
Fish	15	4.97
Cattle	10	3.31
Dogs	9	2.98
Birds (incl. Poultry)	7	2.32
Amphibians, Reptiles	6	1.99
Donkeys, Horses	5	1.66
Pigs	5	1.66
Sheep, Goats	5	1.66
Primates	3	0.99
Rabbits	2	0.66
Cats	1	0.33
Guinea Pigs	1	0.33
Invertebrates	1	0.33
Other Mammals	1	0.33
Other Rodents	1	0.33

### Measures to avoid bias

**Fig 1:**
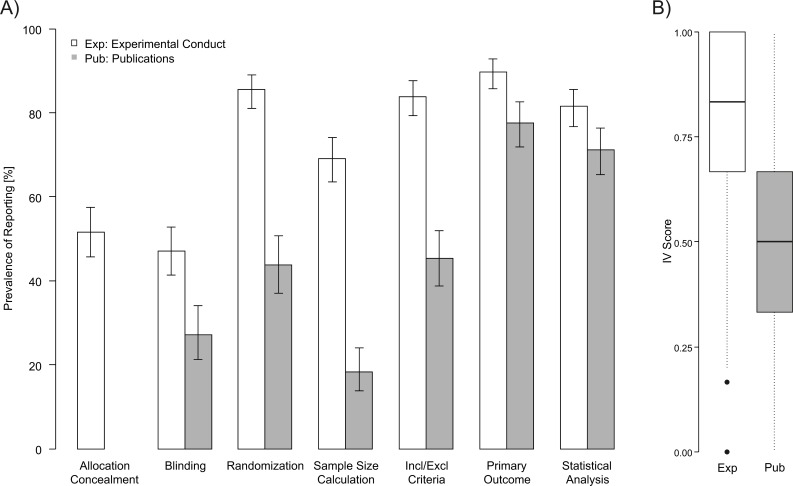
Prevalence of the measures used and reported to avoid risks of bias by the participants to online survey. (A) Prevalence of use of bias avoidance measures during experimental conduct and reporting in the participants’ latest publication (percentages are corrected for ‘no answer’, ‘does not apply to last manuscript’ and ‘have not published so far’. (B) Internal validity scores (IVS) for experimental conduct and reporting in publications.

When asked which of the seven measures against risks of bias the participants normally used in the conduct of their experiments (including the answers ‘yes’ and ‘depends’), a large majority ticked primary outcome variable (90%, N = 264), inclusion and exclusion criteria (84%, N = 245), randomization (86%, N = 248), and statistical analysis plan (82%, N = 240). More than half also ticked sample size calculation (69%, N = 203) and allocation concealment (52%, N = 143), whereas less than half (47%, N = 135) ticked blinded outcome assessment (see Fig 1A white bars). These proportions were corrected for the number of participants ticking ‘no answer’ (for full results including absolute numbers see S2 Table).

To put these numbers in relation to reporting rates derived from published papers, we asked participants to state explicitly which of these seven measures against risks of bias they had reported in their latest published research article. Most of the participants indicated that they had reported in full details a statistical analysis plan (71%, N = 180) and the primary outcome variable (78%, N = 177), whereas reporting rates for inclusion and exclusion criteria (45%, N = 97), randomization (44%, N = 87), sample size calculation (18%, N = 40) and blinding (27%, N = 49) were considerably lower (Fig 1A grey bars). Again, reporting rates were corrected for the number of participants having ticked ‘does not apply to last manuscript’, ‘have not published so far’, and ‘no answer’.

For each of these bias avoidance measures, between 5.3% (statistical analysis) and 27.5% (blinding) of the 302 participants considered these measures to be irrelevant with respect to their latest publication (see “NA” in S2B Table). The most common reason (chosen from a drop-down list) for not reporting measures against risks of bias in their latest publications was that it was ‘not necessary’ (from 30% for sample size calculation up to 80% for statistical analysis). Additional reasons were that it was ‘not common’ (up to 39% for sample size calculation), that they ‘did not think of it’ (up to 19% for primary outcome variable) or space limitations by the journals (up to 8% for sample size calculation).

### IV scores

The mean IVS_Exp_ based on all seven measures against risks of bias was 0.73 (SD ± 0.24, N = 301). However, to facilitate comparison with the IVS_Pub_, we also calculated an IVS_Exp_ based on six measures only (excluding allocation concealment), resulting in a mean IVS_Exp_ of 0.76 (SD± 0.23; N = 301) compared to a mean IVS_Pub_ of 0.49 (SD ± 0.29, N = 261). There was a weak but significant positive correlation between IVS_Exo_ and IVS_Pub_ (Spearman’s Rank Correlation: S = 2322088, Rho = 0.22, p- value = 0.0004).

**Fig 2:**
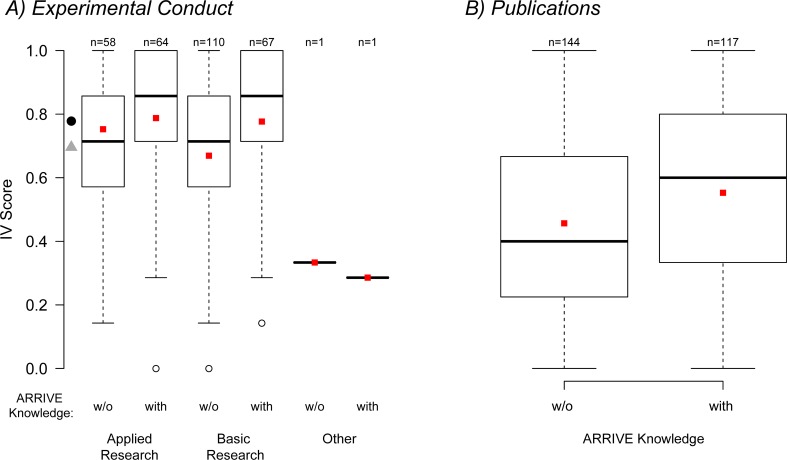
Boxplot of IVS versus descriptors of model selection process. Descriptors are selected for the models with lowest BIC, thus best explaining the variation in IVS for (A) experimental conduct and (B) for publications. For (A) one value is missing as no IVS could be calculated, and for (B) 41 values are missing, because participants ticked ‘have not yet published’, gave ‘no answer’ or declared that these questions ‘do not apply to last manuscript’. For experimental conduct (A), the model including ARRIVE knowledge and research field best explained the IVS_Exp_, whereas for publications (B), the model including only ARRIVE knowledge best explained the IVS_Pub_. Red squares indicate the mean IVS; black circle the mean of IVS_Exp_ of participants with ARRIVE knowledge; grey triangle the mean IVS_Exp_ of participants without ARRIVE knowledge. Whiskers are 1.5*interquartile range.

Variation in IVS_Exp_ was best explained by knowledge of the ARRIVE guidelines and by the participants’ field of research (BIC = 1132, BIC_*weights*_ = 0.967; ΔBIC to second best model [only including ARRIVE knowledge] = 7.38). There was a positive effect of ‘ARRIVE knowledge’ on the IVS_Exp_ compared to ‘no knowledge’ (model estimate = 0.406, 95% CI = 0.133–0.683), and negative effects of ‘basic research’ (model estimate = -0.299, 95% CI = -0.579–-0.0223) and ‘other research’ (model estimate = -2.052, 95% CI = -3.912–-0.526) compared to ‘applied research’ (Fig 2A).

The model including knowledge of the ARRIVE guidelines performed best in explaining variation in the IVS_Pub_ (BIC = 874.24, BIC_*weights*_ = 0.995, ΔBIC to second best model [null model with intercept only] = 11.4). Again, knowledge of the ARRIVE guidelines had a positive effect on the IVS_Pub_ compared to ‘no knowledge’ (model estimate = 0.461, 95% CI = 0.201–0.723) (Fig 2B). An overview of the models and selection procedure can be found in S3 Table.

**Fig 3:**
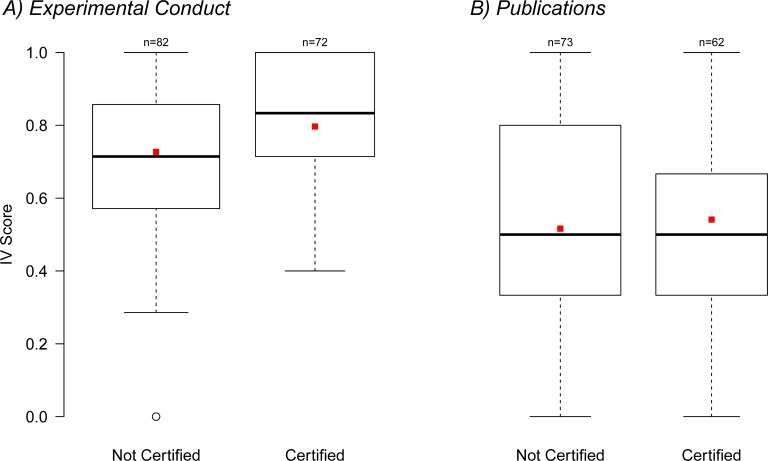
Boxplot of IVS versus certification of institution. Comparison of IVS for (A) experimental conduct, and (B) for the reporting in publications with respect to working institutions being certified. Mean IVS are slightly but non-significantly higher for participants working for certified institutions.

The IVS_Exp_ was slightly higher in participants from certified institutions (mean IVS_Exp_ certified = 0.81, SD ± 0.30, N = 72) compared to non-certified institutions (mean IVS_Exo_ non-certified = 0.73 ± 0.23, N = 82; Fig 3A), however, this difference was not significant (Wilcox Rank Sum Test, W = 2490, p = 0.087). Similarly, IVS_Pub_ was slightly but not significantly higher (W = 2144, p = 0.60) in participants working at certified institutions (mean IVS_Pub_ = 0.54 ± 0.27, N = 62; mean IVS_Pub_ = 0.51 ± 0.30, N = 73; Fig 3B).

### Awareness of risk of bias and measures aimed to avoid them

As summarized in Table 4, most participants indicated that they were aware of risks of bias caused by selective reporting (67.5%, N = 204), selection bias (65.2%, N = 197), and detection bias (61.9%, N = 187), and that they avoid these risks routinely in their research. Furthermore, about half of the participants indicated being aware of publication bias (57.6%, N = 174) and performance bias (48.7%, N = 147), whereas less than one third (29.8%, N = 90) indicated being aware of attrition bias. However, depending on the type of bias only between 15.6% and 41.7% of the participants indicated being concerned about these biases with respect to their own research, and between 15.2% and 35.8% of the participants indicated that these biases did not apply to their own research. Moreover, 10.9% (N = 33) of the participants indicated not being aware of any of these biases, and 24.2% (N = 73) indicated that they were not concerned about any of these biases with respect to their own research (Table 4).

**Table 4:** Participants’ Assessment of Different Types of Experimental Biases.

	A) Awareness	B) Concerned	C) Avoidance	D) Not Relevant
Selective Reporting	67.5 (204)	37.4 (113)	58.9 (178)	17.5 (53)
Selection Bias	65.2 (197)	39.7 (120)	58.3 (176)	17.9 (54)
Detection Bias	61.9 (187)	41.7 (126)	52.3 (158)	15.2 (46)
Publication Bias	57.6 (174)	35.4 (107)	41.7 (126)	24.5 (74)
Performance Bias	48.7 (147)	29.1 (88)	45.7 (138)	22.8 (69)
Attrition Bias	29.8 (90)	15.6 (47)	22.8 (69)	35.8 (108)
None of Above	10.9 (33)	24.2 (73)	13.9 (42)	42.7 (129)
Other Types of Bias	3.0 (9)	1.7 (5)	2.0 (6)	1.7 (5)

Questions included (A) what biases participants were generally aware of, (B) which biases they were concerned with in their own research, (C) which types of bias they were trying to avoid routinely, and (D) which of these biases did not apply to their own research. Shown are percentages with the absolute numbers in brackets.

**Fig 4:**
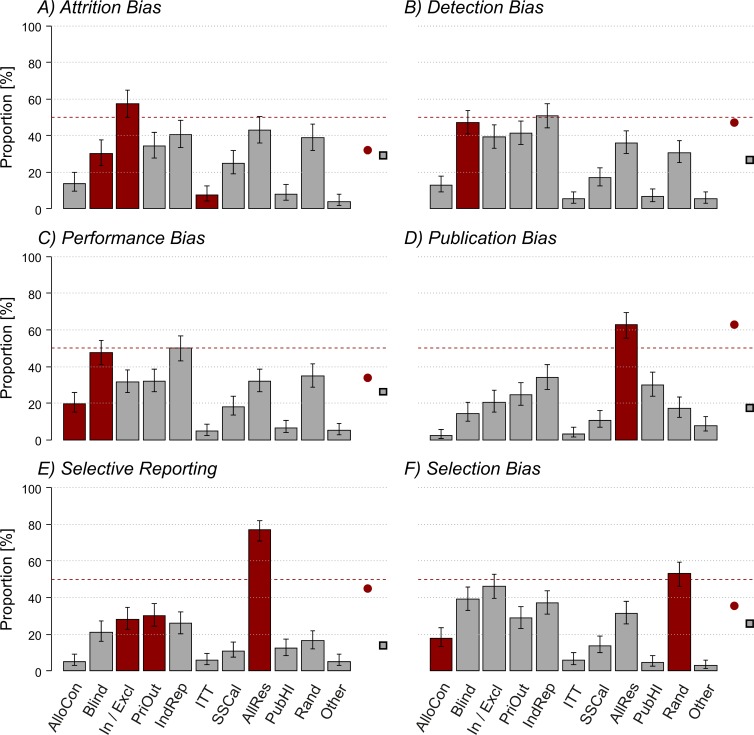
Experimental biases and measures to avoid them. Bars indicate percentage of participants (y-axis) giving that answer (corrected for participants choosing ‘no answer’), red bars indicate effective measures to avoid a given bias (A-F), respectively. The red circle is indicative of the mean of effective measures (*sensu stricto* according to Table 1), while the grey rectangle is the mean of ineffective measures. Number of participants answering to questions: attrition bias N = 172; detection bias N = 224; performance bias N = 212; publication bias N = 180; selective reporting N = 213; selection bias N = 219. The list of possible answers (x-axis) included: AlloCon = allocation concealment; Blind = blinding; In / Excl = inclusion / exclusion criteria; PriOut = primary outcome variable; IndRep = independent replication; ITT = Intention-to-Treat analysis; SSCal = sample size calculation; AllRes = reporting of all results; PubHI = publishing in high impact journals; Rand = randomization; Other = other measures.

Next, we assessed the participants’ knowledge of specific measures against risks of bias. Fig 4 presents their responses when asked what measures they would take to avoid the different types of bias. As indicated by the distribution of responses across the different panels, apart from publication bias (panel D), there was no clear pattern of preference for effective over ineffective measures.

**Fig 5:**
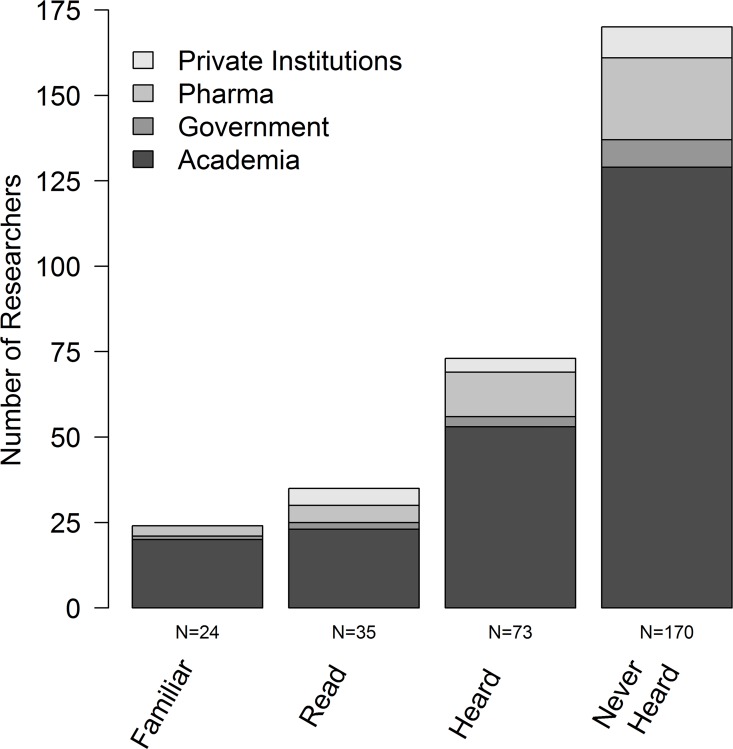
Knowledge of ARRIVE Guidelines by participants to the online survey. Shown are absolute numbers of participants being ‘familiar’ with the guidelines, ‘having read’ and ‘having heard of’ them, and having ‘never heard of’ the guidelines, split according to the participants’ affiliations (private institutions, pharma, governmental institutions and academia).

The ARRIVE guidelines were known by 43.7% of the participants (N = 132), of which 24 indicated that they were familiar with these guidelines, 35 that they had read them, and 73 that they had heard of them. However, the majority of participants (56.3%, N = 170) indicated that they had never heard of the ARRIVE guidelines before (Fig 5). Among the 132 participants being aware of the ARRIVE guidelines, most indicated that they adhere to them either generally (30.3%, N = 40) or occasionally 34.8%, N = 46), while 15.2% (N = 20) answered that they did not adhere to them and 19.7% (N = 26) did not answer this question.

Consulting the NC3rs Website (https://www.nc3rs.org.uk/arrive-animal-research-reporting-vivo-experiments#journals, accessed July 6^th^ 2015), the journals in which participants had published their latest paper was checked for endorsement of the ARRIVE guidelines. Of all participants having published at least one research paper (N = 288), 79 (27.4%) had published their latest paper in a journal that had endorsed the ARRIVE guidelines (86.1% [N = 68] from academia, 6.3% [N = 5] from governmental institutions, 5.1% [N = 4] from industry, 2.5% [N = 2] from private research institutions). Among these participants, 16.5% (N = 13) indicated that they were familiar with the ARRIVE guidelines, 11.4% (N = 9) that they had read them, and 21.5% (N = 17) that they had heard of them. However, more than half of the participants who had last published in a journal endorsing the ARRIVE guidelines (51%, N = 40) indicated that they had never heard of these guidelines.

Apart from the ARRIVE guidelines, 235 participants (77.8%) indicated that they adhered to other guidelines either regularly (N = 217, 71.8%) or occasionally (N = 18, 6%), while 26 participants (8.6%) never followed any guidelines (41 participants [13.6%] did not answer this question). Among the 235 participants who did adhere to other guidelines either regularly or occasionally, 72.3% (N = 170) referred to internal SOPs (Standard Operating Procedure), 59.6% (N = 140) to specific journal guidelines, and 24.2% (N = 57) to various other guidelines (multiple answers were possible).

### Assessment of possible bias in study sample

To assess whether our study sample of fully completed questionnaires (N = 302; 16% of total survey population) might be biased, we exploited our sample of partially completed questionnaires (N = 228) and compared participant characteristics between these two samples as well as the primary outcome variable of this study, IVS_Exp_.

Similar proportions of participants returning partially vs. fully completed questionnaires ascribed their research to basic research (51.2% vs. 58.9%), applied research (45.5% vs. 40.4%), or were undecided (2.4% vs. 0.7%), respectively (N = 167 partially completed questionnaires). Also, similar proportions were involved in biomedical and medical research (85.6% vs. 86.8%; N = 167); were using mice (71.5% vs. 60.6%) and rats (13.9% vs. 15.6%; N = 165); and were affiliated with academic institutions (71.1% vs. 74.5%), industry (15.1% vs. 14.9%), governmental research institutions (5.7% vs. 4.6%), and private research institutions (8.2% vs. 6%; N = 159), respectively.

In terms of the primary outcome variable, IVS_Exp_, the comparison between our study sample of fully completed questionnaires and that of partially completed questionnaires for which the necessary answers were available yielded identical results, with a mean IVS_Exp_ of 0.73 (SD ± 0.24; N = 301) for the study sample and a mean IVS_Exp_ of 0.73 (SD ± 0.20; N = 99) for the sample of partially completed questionnaires.

## Discussion

### Summary of results

Low reporting rates of measures against risks of bias in the primary literature are widely considered as a proxy measure of poor experimental conduct. Reporting guidelines (e.g., ARRIVE) have thus become a major weapon in the fight against risks of bias in animal research. Here we studied, for the first time, how reporting rates of measures against risks of bias in in vivo research (e.g. [23,26,28–30,33]) relate to the rates at which such measures are implemented, according to researchers’ self-reports. Our findings indicate that scientific rigor of animal research may be considerably better than predicted by reporting rates, as researchers may be using measures against risks of bias to a much greater extent than suggested by systematic reviews of the published literature. The large discrepancy suggests that reporting rates may be poor predictors of scientific rigor in animal research. This is further supported by our finding that the rates at which researchers claimed to have reported measures against bias in their latest publication were considerably lower than the rates at which they claimed to have used these measures in their research.

On the other hand, we found a weak but positive correlation between self-reported use and self-reported reporting of measures against risks of bias, supporting findings from systematic reviews indicating that higher reporting rates reflect more rigorous research (e.g. [26,29,48]). Furthermore, self-reported reporting rates of measures against risks of bias in the researchers’ latest publication were considerably higher than the reporting rates commonly found by systematic reviews. Taken together, these findings suggest that whereas reporting rates may underestimate scientific rigor, self-reports may overestimate it. The latter is further supported by our finding that the researchers’ knowledge of risks of bias, and effective measures to prevent them, was rather limited. Thus, the discrepancy between reporting rates and self-reports may be partly explained by the researchers’ ignorance of potential risks of bias and measures to prevent them.

Our findings, therefore, highlight a need for better education and training of researchers in good research practice to raise their awareness of risks of bias and improve their knowledge about measures to avoid them. Furthermore, they indicate a need for more reliable predictors of scientific rigor.

### Validity of self-reports

The researchers’ self-reports of their use of measures against risks of bias should be interpreted with caution, as self-reports may not necessarily reflect the true quality of experimental conduct. That the reporting rates of measures against bias claimed by the researchers for their latest publication were considerably higher than the reporting rates generally found by systematic reviews of the published literature (e.g., randomization 44% vs. 27%, sample size calculation 18% vs. 0.5%) indicates that the researchers’ self-reports should not be taken at face value. There are two main ways in which the self-reports may be biased. First, our study population (participants having returned fully completed questionnaires) may differ from the overall population of in vivo researchers. For example, participants of the survey may be particularly conscious of risks of bias and the problem of poor reproducibility, which may have predisposed them to take part in this survey. This could explain better experimental conduct and better reporting, compared to the overall population. Alternatively, participants may have been prone to overestimate their own performance (e.g. [49]). We have only limited data to assess these two alternatives. However, when comparing the population of participants who returned fully completed questionnaires with the population of participants who started but did not complete the questionnaire, we did not find any major differences in the characteristics of the participants (e.g., host institution, research animals, type of research), nor in the primary outcome variable of this study, the internal validity score for experimental conduct (IVS_Exp_). Given that these two populations of participants together accounted for almost one third of the overall population of registered in vivo researchers in Switzerland, the difference in reporting rates between self-reports and systematic reviews are unlikely to be explained by a systematic bias towards better performers in our study sample. This is further supported by the fact that the participants performed rather poorly when asked about their knowledge of specific types of bias, and effective measures to avoid these. Overestimation of one’s own performance tends to be the more pronounced, the less skilled and competent individuals are (i.e., the Kruger-Dunning Effect, [50]). Although researchers are generally highly skilled and competent in their field of research, the researchers’ limited knowledge of types of bias and measures to avoid them renders their self-reports at risk for overestimation. We thus conclude that the difference between what researchers claimed to have reported in their latest paper and reporting rates found by systematic reviews are more likely explained by the researchers overestimating their own performance than a bias towards better performers in our study sample.

Subjective bias resulting in overestimation of their own performance may also have affected the researchers’ self-reports on the actual use of measures against risks of bias. Thus, the true use of measures against risks of bias may lie anywhere between what has been found to be reported by systematic reviews, and the researchers’ self-report presented here. Given the large difference between IVS_Exp_ and IVS_Pub_, however, reporting rates found in the literature are likely to underestimate scientific rigor to a considerable extent.

### Reasons for low reporting rates

The main reason for not reporting the use of measures against risks of bias in publications is that researchers do not find it necessary to report it. This was further corroborated by personal interviews. Thus, researchers argued, for example, that “certain things are self-evident and do not need to be reported”, that “the journal did not request to describe it [e.g., randomization]”, that “good scientific practice” actually implies that the criteria of good research practice are met without having to stress (i.e., report) this, or that “there is a threshold for what is relevant to the own laboratory and [what is relevant] to the research community outside the laboratory”.

However, given the negative relationship between the reporting of measures against risks of bias and overstatement of treatment effect size (e.g. [28,29,30,40]), and the positive correlation between IVS_Pub_ and IVS_Exp_ found here, these statements appear questionable.

Although our findings suggest that scientific rigor in animal research may be considerably better than predicted by systematic reviews, there clearly is scope for improvement as, for example, only half of the participants self-reported using blinded outcome assessment (47%) or allocation concealment (52%). Blinding and allocation concealment, together with proper randomization procedures, are key measures to avoid selection bias and detection bias (cf. Table 1) and should be used in every study and reported in every publication (e.g. [5,31,51]).

### Effect of knowledge of reporting guidelines on measures of scientific rigor

To assess the effects of specific characteristics of the researchers or their research on measures of scientific rigor, we calculated scores of experimental conduct (IVS_Exp_) and reporting (IVS_Pub_). Similar scores have previously been used to assess scientific rigor in systematic reviews and meta-analyses of reporting rates in the published literature (e.g., CAMARADES checklist [24]). Variation in IVS_Exp_ was best explained by knowledge of the ARRIVE guidelines (yes vs. no) and type of research (applied vs. basic vs. other). Thus, researchers being familiar with the ARRIVE guidelines and researchers in applied research scored higher on IVS_Exp_, and researchers knowing the ARRIVE guidelines also scored higher on IVS_Pub_. These findings support the view that reporting guidelines may improve not only reporting but may actually improve the use of measures against risks of bias (e.g. [48]). The positive effect of applied research on IVS_Exp_ is more difficult to explain. It has previously been argued that the incentive for reliable results may be higher in applied research, for example in pharma research where also economic values are at stake (e.g. [19,52]). However, given the small size of this effect, and the fact that participants from academia and industry did not differ on both scores (IVS_Exp_: academia = 0.73 vs. industry = 0.73, Wilcox test: W = 5243, p = 0.70; IVS_Pub_: academia = 0.51 vs. industry = 0.46, Wilcox test: W = 3900, p = 0.40) suggests that it should be interpreted with caution.

Despite loud calls for better reporting (e.g. [53]) and the widespread endorsement of reporting guidelines by many scientific journals (e.g. [34,54–56]), reporting has not yet improved much [35]. Thus, without active enforcement of reporting guidelines by journal editors and reviewers, the situation may not change [57]. This is also confirmed by results of this study: more than half of the participants having published their latest article in a journal that has endorsed the ARRIVE guidelines admitted that they had never heard of these guidelines. This ignorance is surprising given the wide coverage that the ARRIVE guidelines have received and we may only speculate about the reason for this. Most likely, researchers can still ignore them–and may continue to do so–as long as the journals do not enforce them more strictly.

This may reflect a general attitude we observed among the scientists we interviewed. While they agreed that guidelines for the design and conduct of experiments may be useful, they were skeptical towards reporting guidelines. As one interviewee put it, “introducing more checklists to tick boxes does not increase the quality of science”. Thus, publication checklists are perceived as a sign of increasing over-regulation and bureaucracy and may therefore be ignored. Similarly, Begley and Ioannidis [39] warned that the burden of bureaucracy might lead to normative responses without measurable benefits for the quality of research and reproducibility. However, Minnerup and colleagues [48] recently showed that the quality of research published in the journal *Stroke* increased after the implementation of the ‘Basic Science Checklist’. Thus, if enforced by reviewers and editors, adequate checklists may well be conductive to the quality of research.

### Knowledge of risks of bias and measures to avoid them

Increasing evidence of bias associated with poor experimental conduct and reporting (e.g. [5,23,26,29,30,33]) is only partly mirrored by the participants’ answers to the questionnaire. Thus, only about two thirds of the participants (58–68%) indicated being aware of selective reporting, selection bias, detection bias, and publication bias, and less than half of them were actually ‘concerned’ about such biases (35–42%) with respect to their own research. Furthermore, between 15% and 25% indicated that these biases were ‘not relevant’ to their own research. These results reflect a certain ignorance of risks of bias in experimental conduct, combined with a lack of knowledge about these risks and about effective measures to avoid them. Thus, when participants were asked about effective measures against specific types of bias from a list of 10 potential measures, there was no consistent preference of effective over ineffective measures, except for publication bias (and, to some extent, for selective reporting). In particular, participants performed poorly when asked for measures against attrition bias, detection bias, and performance bias, respectively. This lack of understanding may have contributed to the participants overestimating the quality of their own experimental conduct. Therefore, besides the implementation of reporting guidelines (e.g. [34,48,56,58,59]), which will raise awareness of risks of bias, we conclude that researchers may need better training in scientific integrity and good research practice in view of minimizing risks of bias in future research.

### Conclusions

Our findings indicate that reporting rates of measures against risks of bias may not be reliable measures of scientific rigor in animal research, and that better measures are needed. However, although the researchers’ self-reports suggest that the actual use of measures against risks of bias may be considerably higher than predicted by the low reporting rates in the published literature, self-reports may overestimate their true use. Indeed, the results presented here indicate that there may be considerable scope for improvement of scientific rigor in experimental conduct of animal research, and that concepts and methods of good research practice should play a more important role in the education of young researchers (e.g. [11]). It is quite possible that lack of scientific rigor contributes to the so called “reproducibility crisis” (e.g. [3]). However, scientific rigor in experimental conduct is not the only factor affecting reproducibility, and perhaps not even the most important one; poor construct validity of animal models (e.g. [9,17]) and poor external validity due to highly standardized laboratory conditions (e.g. [8,60–63]) are important alternative causes. Further research is therefore needed on the effects of different aspects of scientific validity on reproducibility, to assess their scope for improvement and in view of prioritizing strategies towards improvement beyond reporting guidelines.

## Supporting Information

S1 Table Overview of Certifications of Participants’ Institutions. (DOCX)

S2 Table Full Results of Use and Reporting of Measures to Avoid Risk of Bias. (DOCX)

S3 Table A) Overview of Candidate Models and B) Model Outputs of Best Performing Models.
(DOCX)

S1 Text Online Survey. (DOCX)

S2 Text Personal Interviews. (DOCX)
