# Supplementary material for: The Researchers’ View of Scientific Rigor—Survey on the Conduct and Reporting of In Vivo Research
Source: PLoS One. 2016 Dec 2;11(12):e0165999. doi: 10.1371/journal.pone.0165999 (PMC5135049; doi:10.1371/journal.pone.0165999)
Supplement: S1 Table — (DOCX) [file pone.0165999.s001.docx]

**S1 Table. Overview of Certifications of Participants’ Institutions**

Participants of the survey were asked whether their institution was certified. In case they ticked ‘yes', sub-questions followed with additional answer options (ISO: ISO 9001, ISO 17025, ISO unknown, ISO others; AAALAC; GLP; GMP; other; see S1 Text questions B4, B5). Numbers in the table are percentages, with absolute numbers in brackets.

|  |  | **Yes** | | **No** | | **Do not know** | | **No Answer** | |
| --- | --- | --- | --- | --- | --- | --- | --- | --- | --- |
| Certified (N=302) | | 23.8 | (72) | 27.2 | (82) | 44.7 | (135) | 4.3 | (13) |
|  |  |  |  |  |  |  |  |  |  |
| If certified (N=72) | |  |  |  |  |  |  |  |  |
|  | ISO | 41.7 | (30) | 58.3 | (42) |  |  |  |  |
|  | AAALAC | 30.6 | (22) | 69.4 | (50) |  |  |  |  |
|  | GLP | 37.5 | (27) | 62.5 | (45) |  |  |  |  |
|  | GMP | 19.4 | (14) | 80.6 | (58) |  |  |  |  |
|  | *other:* OLAW | 1.4 | (1) |  |  |  |  |  |  |
|  |  |  |  |  |  |  |  |  |  |
| If ISO certified (N=30) | |  |  |  |  |  |  |  |  |
|  | ISO 9001 | 16.7 | (5) | 83.3 | (25) |  |  |  |  |
|  | ISO 17025 | 43.3 | (13) | 56.7 | (17) |  |  |  |  |
|  | ISO unknown | 56.7 | (17) |  |  |  |  |  |  |
|  | *other:* ISO 17020 | 3.3 | (1) |  |  |  |  |  |  |

ISO: International Organization for Standardization, <http://www.iso.org/iso/home/search.htm>

AAALAC: Association for Assessment and Accreditation of Laboratory Animal Care, <http://www.aaalac.org/accreditation/>

GLP: Good Laboratory Practice, <http://www.oecd.org/chemicalsafety/testing/goodlaboratorypracticeglp.htm>

GMP: Good Manufacturing Practice, <http://www.ispe.org/gmp-resources>

OLAW: *Office of Laboratory Welfare,* <http://grants.nih.gov/grants/olaw/olaw.htm>
