# Supplementary material for: The Researchers’ View of Scientific Rigor—Survey on the Conduct and Reporting of In Vivo Research
Source: PLoS One. 2016 Dec 2;11(12):e0165999. doi: 10.1371/journal.pone.0165999 (PMC5135049; doi:10.1371/journal.pone.0165999)
Supplement: S2 Table — (DOCX) [file pone.0165999.s002.docx]

**S2 Table. Full Results of Use and Reporting of Measures to Avoid Risks of Bias**

(A) Measures to avoid risks of bias used during experimental conduct; (B) measures to avoid risks of bias reported in the latest published manuscript; (C) reasons for not reporting measures to avoid risks of bias the latest published manuscript.
Numbers give absolute values, while the percentage of the total sample (N=302) is given in brackets.

| ***A) Measures to Avoid Risks of Bias During Experimental Conduct*** | | | | | | | | | | | | |
| --- | --- | --- | --- | --- | --- | --- | --- | --- | --- | --- | --- | --- |
|  | Yes* | | No* | | Not Sure* | | Depends* | | No Answer | |  |  |
| Sample Size Calculation | 163 | (54.0) | 83 | (27.5) | 8 | (2.6) | 40 | (13.2) | 8 | (2.6) |  |  |
| Statistical Analysis Plan | 221 | (73.2) | 47 | (15.6) | 7 | (2.3) | 19 | (6.3) | 8 | (2.6) |  |  |
| Inclusion & Exclusion Criteria | 238 | (78.8) | 41 | (13.6) | 6 | (2.0) | 7 | (2.3) | 10 | (3.3) |  |  |
| Blinded Outcome Assessment | 92 | (30.5) | 144 | (47.7) | 8 | (2.6) | 43 | (14.2) | 15 | (5.0) |  |  |
| Randomization | 226 | (74.8) | 34 | (11.3) | 8 | (2.6) | 22 | (7.3) | 12 | (4.0) |  |  |
| Allocation Concealment | 119 | (39.4) | 118 | (39.1) | 16 | (5.3) | 24 | (7.9) | 25 | (8.3) |  |  |
| Primary Outcome Variable | 255 | (84.4) | 18 | (6.0) | 12 | (4.0) | 9 | (3.0) | 8 | (2.6) |  |  |

| ***B) Measures to Avoid Risks of Bias Reported in the Latest Publication*** | | | | | | | | | | | | |
| --- | --- | --- | --- | --- | --- | --- | --- | --- | --- | --- | --- | --- |
|  | Yes  [+*‘some details’*] | | No* | | Not Remember* | | NA | | Not Published | | No Answer | |
| Sample Size Calculation | 40* [35] | (24.8) | **143** | (47.4) |  |  | 51 | (16.9) | 24 | (7.95) | 9 | (3.0) |
| Statistical Planning | 180* [68] | (82.1) | **5** | (1.7) |  |  | 16 | (5.3) | 23 | (7.6) | 10 | (3.3) |
| In- & Exclusion Criteria | 97* [60] | (52.0) | **57** | (18.9) |  |  | 54 | (17.9) | 24 | (7.9) | 10 | (3.3) |
| Information on Blinding | **49*** | (16.2) | 122 | (40.4) | 9 | (3.0) | 83 | (27.5) | 30 | (9.9) | 9 | (3.0) |
| Randomization | 87* | (28.8) | **105** | (34.8) | 7 | (2.3) | 62 | (20.5) | 30 | (9.9) | 11 | (3.6) |
| Allocation Concealment | 110 | (36.4) | **66** | (21.9) | 9 | (3.0) | 65 | (21.5) | 30 | (9.9) | 22 | (7.3) |
| Primary Outcome Variable | 177* | (58.6) | **43** | (14.2) | 8 | (2.6) | 31 | (10.3) | 30 | (9.9) | 13 | (4.3) |

| ***C) Reasons for Non-Reporting in Latest Publication*** | | | | | | | | | | | | |
| --- | --- | --- | --- | --- | --- | --- | --- | --- | --- | --- | --- | --- |
|  | NA | | Did Not Think About It | | Not Common | | Not Necessary | | Journal Space Limitations | | Other Reasons | |
| Sample Size Calculation (N=143) |  |  | 7 | (4.9) | 55 | (38.5) | 43 | (30.1) | 11 | (7.7) | 27 | (18.9) |
| Statistical Planning (N=5) |  |  |  |  |  |  | 4 | (80.0) |  |  | 1 | (20.0) |
| In- & Exclusion Criteria (N=57) |  |  | 7 | (12.3) | 14 | (24.6) | 26 | (45.6) | 2 | (3.5) | 8 | (14.0) |
| Randomization (N=105) | 9 | (8.6) | 14 | (13.3) | 28 | (26.7) | 41 | (39.0) | 6 | (5.7) | 7 | (6.7) |
| Allocation Concealment (N=66) | 22 | (33.3) | 3 | (4.5) | 9 | (13.6) | 21 | (31.8) | 2 | (3.0) | 9 | (13.6) |
| Primary Outcome Variable (N=43) | 7 | (16.3) | 8 | (18.6) | 10 | (23.3) | 14 | (32.6) | 2 | (4.7) | 2 | (4.7) |

**Notes on table entries:**

**:* Used to calculate the internal validity score IVS

*Yes*: Measure used or reported

*No*: Measure not used or not reported

*Not sure*: Participant was not sure about whether measure was used

*Not remember*: Participant did not remember whether measure was reported

*Depends*: Need for use and reporting of measures may sometimes depend on the specific study (if this option was ticked, more info was requested in a follow up question)

*NA*: Participant was of the opinion that the measure was irrelevant or not applicable to the latest publication

*Not published*: Participant has not published so far

*No answer*: Participants chose not to answer this question

*Did not think about it*: Participant did not think about reporting this measure

*Not common*: According to participant, reporting of this measure is not common practice

*Not necessary*: Participant thought it was not necessary to report this measure

*Journal space limitation:* Space limitation given by journal / publisher did not allow reporting of this measure

*Other reasons*: Comment box for additional reasons why certain measures were not reported

*Unknown*: Status of blinding was not known by participant

*NA*: Participant was not involved in experiment / Participant was not in a certain role for the experiment
