# Supplementary material for: The Researchers’ View of Scientific Rigor—Survey on the Conduct and Reporting of In Vivo Research
Source: PLoS One. 2016 Dec 2;11(12):e0165999. doi: 10.1371/journal.pone.0165999 (PMC5135049; doi:10.1371/journal.pone.0165999)
Supplement: S3 Table — A) Overview of Candidate Models and B) Model Outputs of Best Performing Models. (DOCX) [file pone.0165999.s003.docx]

| **A) Candidate Models**  **S3 Table. A) Overview of Candidate Models** | **Experience** | **ARRIVE** | **Institution** | **Canton** | **Res - Area** | **Res - Field** | **BIC** | **delta BIC** | **BIC weights** |  |
| --- | --- | --- | --- | --- | --- | --- | --- | --- | --- | --- |
| ***i) Design and Conduct*** | |  |  |  |  |  |  |  |  |  |
| ***Model 6*** |  | **+** |  |  |  | **+** | 1131.999 | 0 | 0.967 |  |
| Model 7 |  | + |  |  |  |  | 1139.376 | 7.377 | 0.024 |  |
| Model 8 |  |  |  |  |  | + | 1141.751 | 9.752 | 0.007 |  |
| Model 5 |  | + |  |  | + | + | 1144.746 | 12.747 | 0.002 |  |
| Model 4 |  | + | + |  | + | + | 1148.9 | 16.901 | 0.000 |  |
| Null-Model *(intercept only)* |  |  |  |  |  |  | 1152.167 | 20.168 | 0.000 |  |
| Model 9 |  |  |  |  | + |  | 1152.983 | 20.984 | 0.000 |  |
| Model 10 | + |  |  |  |  |  | 1154.879 | 22.88 | 0.000 |  |
| Model 3 |  | + | + |  | + | + | 1159.614 | 27.615 | 0.000 |  |
| Model 11 |  |  | + |  |  |  | 1169.047 | 37.048 | 0.000 |  |
| Model 2 | + | +* | +* |  | + | + | 1172.503 | 40.504 | 0.000 |  |
| Model 12 |  |  |  | + |  |  | 1203.567 | 71.568 | 0.000 |  |
| Model 1 | + | +* | +* | + | + | + | 1230.212 | 98.213 | 0.000 |  |
|  |  |  |  |  |  |  |  |  |  |  |
| ***ii) Reporting*** |  |  |  |  |  |  |  |  |  |  |
| ***Model 7*** |  | **+** |  |  |  |  | 874.238 | 0.000 | 0.995 |  |
| Null-Modell *(intercept only)* |  |  |  |  |  |  | 885.628 | 11.389 | 0.003 |  |
| Model 10 | + |  |  |  |  |  | 888.785 | 14.546 | 0.001 |  |
| Model 6 |  | + |  |  | + |  | 889.275 | 15.036 | 0.001 |  |
| Model 5 |  | + |  |  | + | + | 892.126 | 17.888 | 0.000 |  |
| Model 8 |  |  |  |  |  | **+** | 892.362 | 18.124 | 0.000 |  |
| Model 9 |  |  |  |  | + |  | 893.014 | 18.776 | 0.000 |  |
| Model 4 | + | + |  |  | + | + | 897.021 | 22.783 | 0.000 |  |
| Model 11 |  |  | + |  |  |  | 900.848 | 26.609 | 0.000 |  |
| Model 3 | + | + | + |  | + | + | 912.104 | 37.865 | 0.000 |  |
| Model 2 | + | +* | +* |  | + | + | 925.804 | 51.566 | 0.000 |  |
| Model 12 |  |  |  | + |  |  | 928.071 | 53.833 | 0.000 |  |
| Model 1 | + | +* | +* | + | + | + | 966.817 | 92.578 | 0.000 |  |
| * indicates interactions between the two variables ‘ARRIVE knowledge’ and ‘institutions’ | | | | | | | | | | |

**S3 Table. B) Model Outputs of Best Performing Models**

Model estimates of best performing models (according to model selection procedure), corrected for overdispersion of the data. The estimate of the intercept modelling the IVS_Exp_ is given for ‘no knowledge of ARRIVE guidelines’ and ‘basic research’, while the estimate of the intercept modelling the IVS_Pub_ stands for ‘no knowledge of ARRIVE guidelines’.

| **B) Model Output** | **Estimate** | **Std. Error** | **t-Value** | **P-Value** |  |
| --- | --- | --- | --- | --- | --- |
| ***Design & Conduct*** (Model 6) | |  |  |  |  |
| *Intercept* | 0.714 | 0.099 | 7.250 | 0.000 | ******* |
| ARRIVE Knowledge (yes) | 0.407 | 0.140 | 2.899 | 0.004 | ****** |
| Applied Research | 0.300 | 0.142 | 2.110 | 0.036 | ***** |
| Other Research | -1.753 | 0.818 | -2.144 | 0.033 | ***** |
|  |  |  |  |  |  |
| ***Reporting*** (Model 7) |  |  |  |  |  |
| *Intercept* | -0.261 | 0.090 | -2.899 | 0.00406 | ****** |
| ARRIVE Knowledge (yes) | 0.461 | 0.133 | 3.472 | 0.0006 | ******* |
| Significance Level: *** < 0.001, ** < 0.01, * < 0.05 | | | | | |
