# Supplementary material for: The Researchers’ View of Scientific Rigor—Survey on the Conduct and Reporting of In Vivo Research
Source: PLoS One. 2016 Dec 2;11(12):e0165999. doi: 10.1371/journal.pone.0165999 (PMC5135049; doi:10.1371/journal.pone.0165999)

**S1 Text. Online Survey**

The following is a copy of the online questionnaire sent out to the 1891 potential participants. It was returned by 530 participants, of which 302 returned fully completed questionnaires. Only fully completed questionnaires were analyzed further, whereas partially completed questionnaires were used for assessing a potential bias in the study sample only.


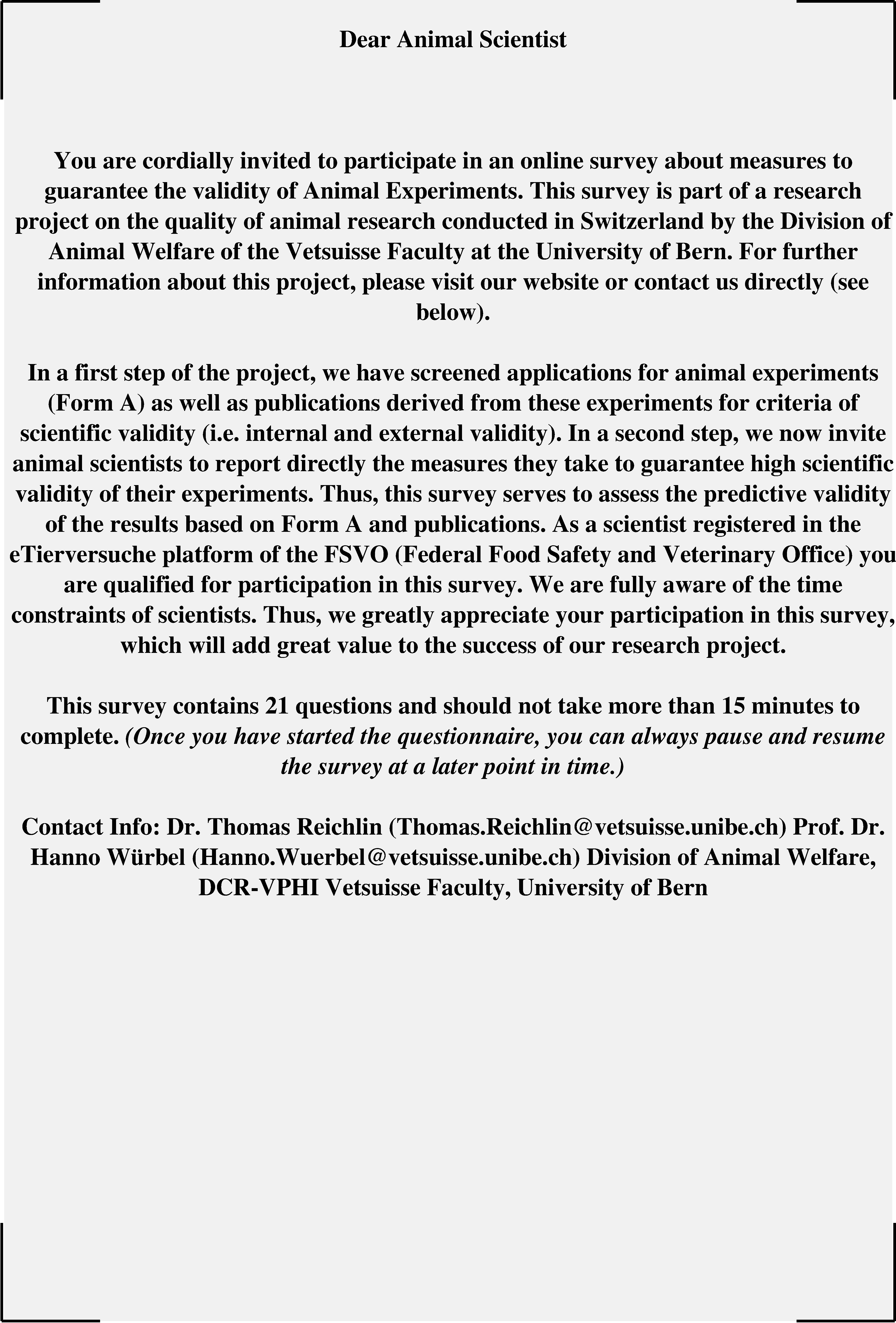


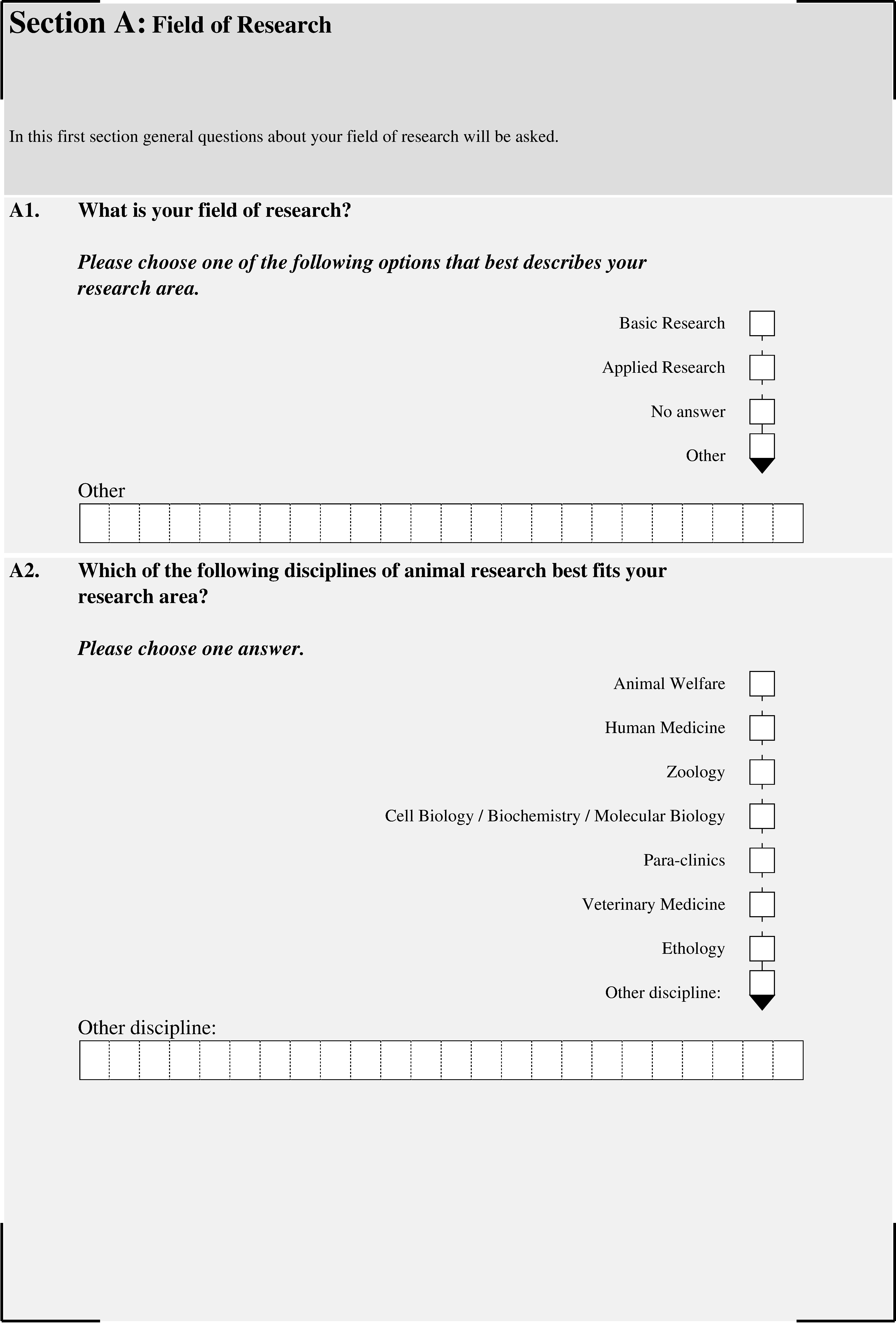


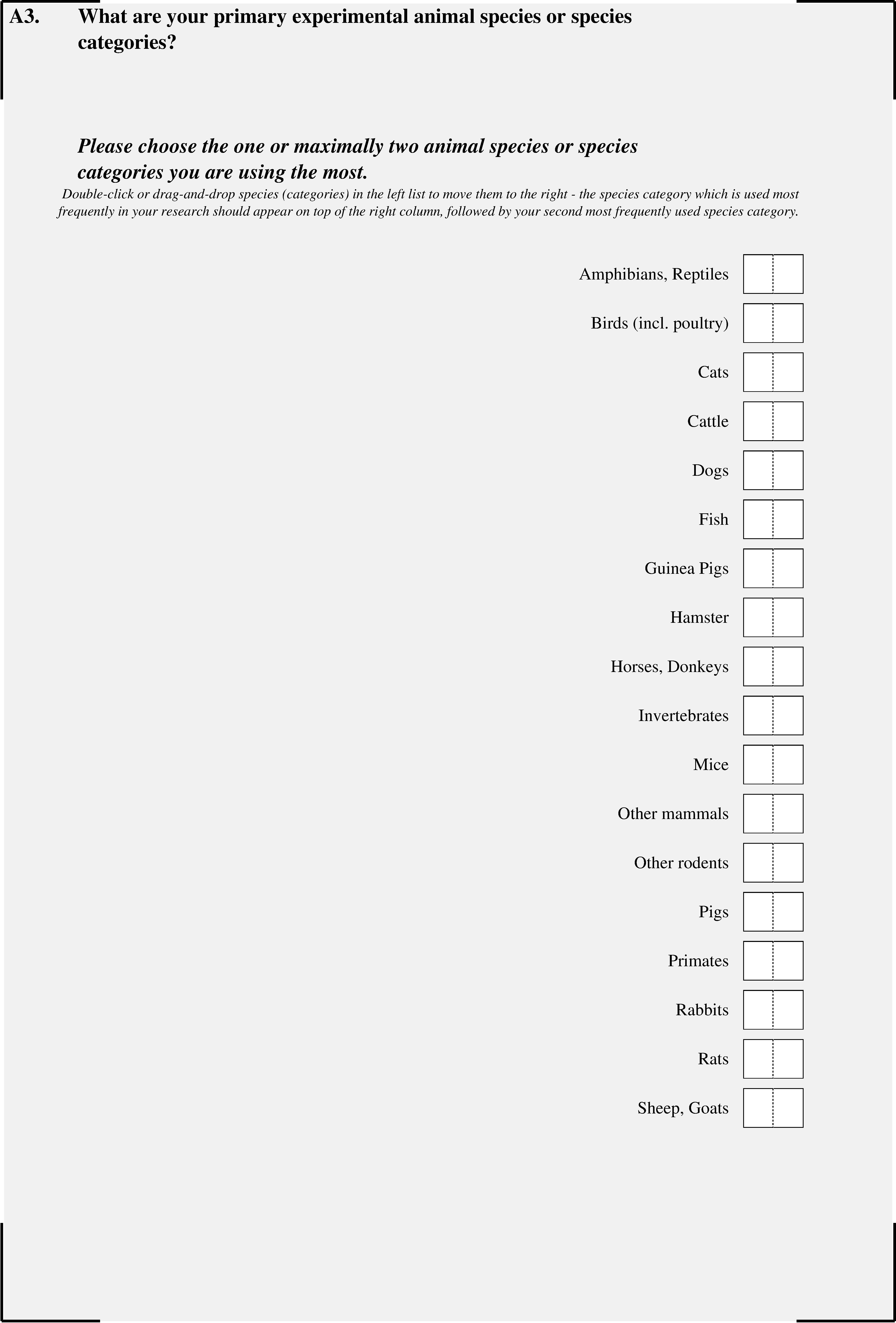


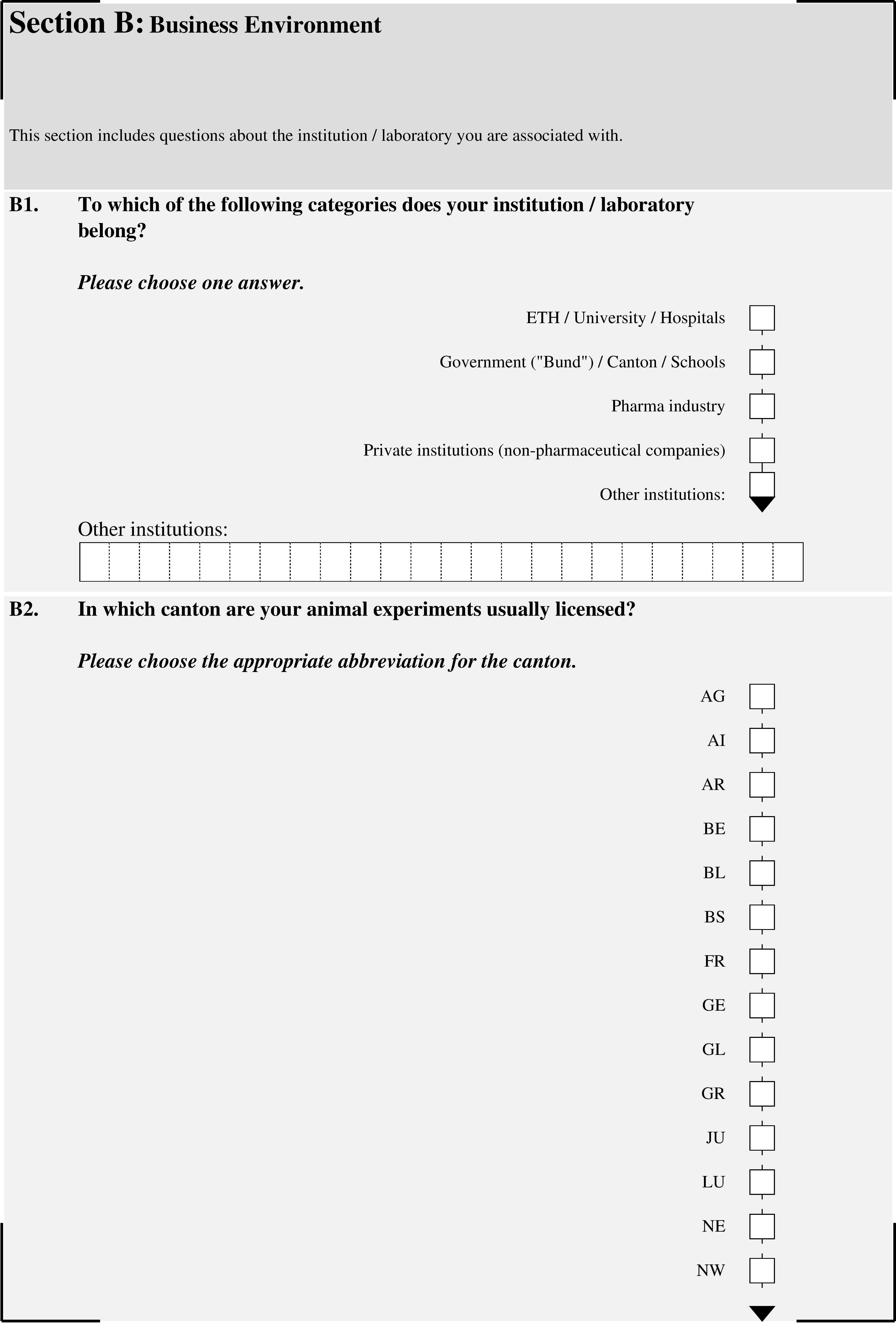


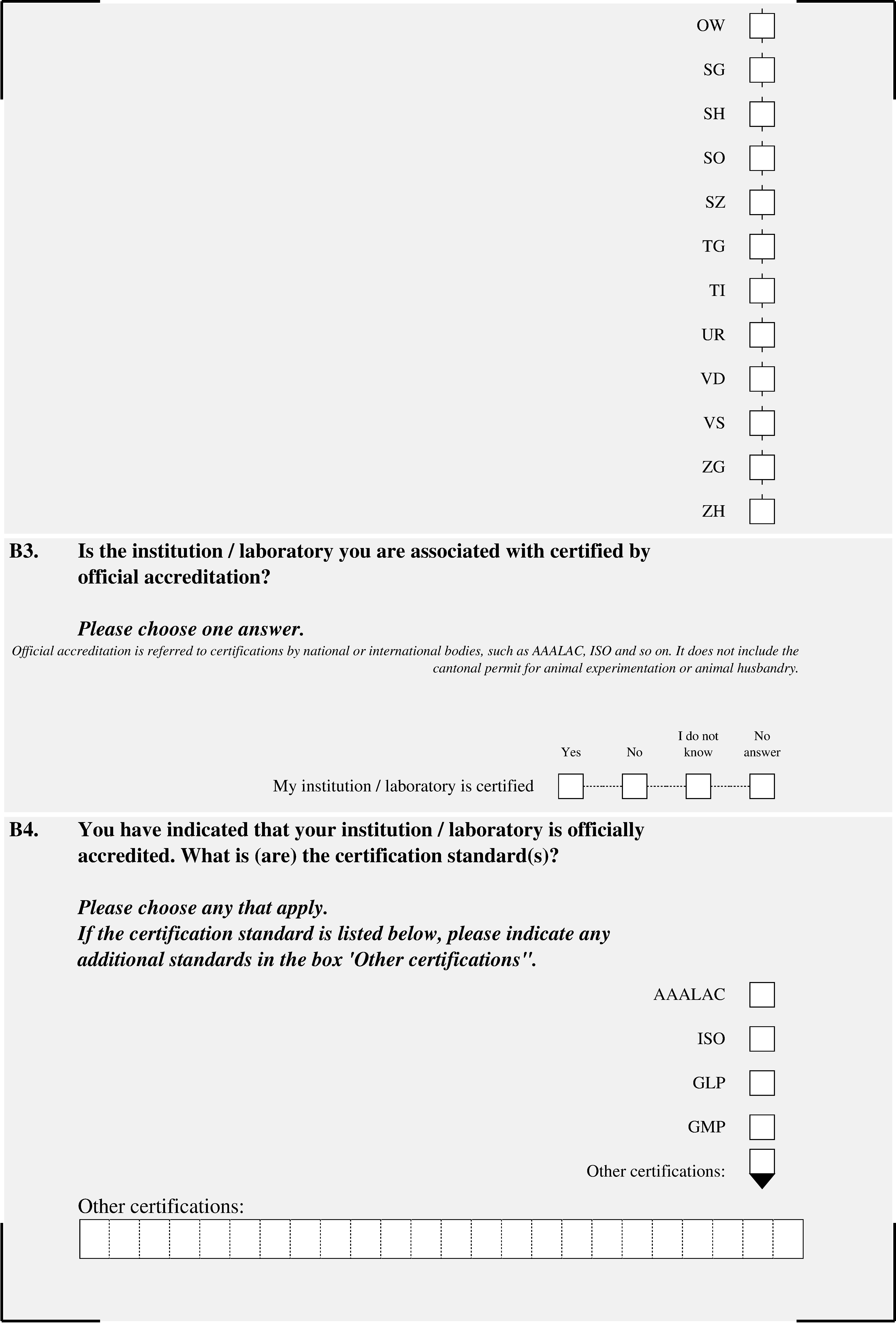


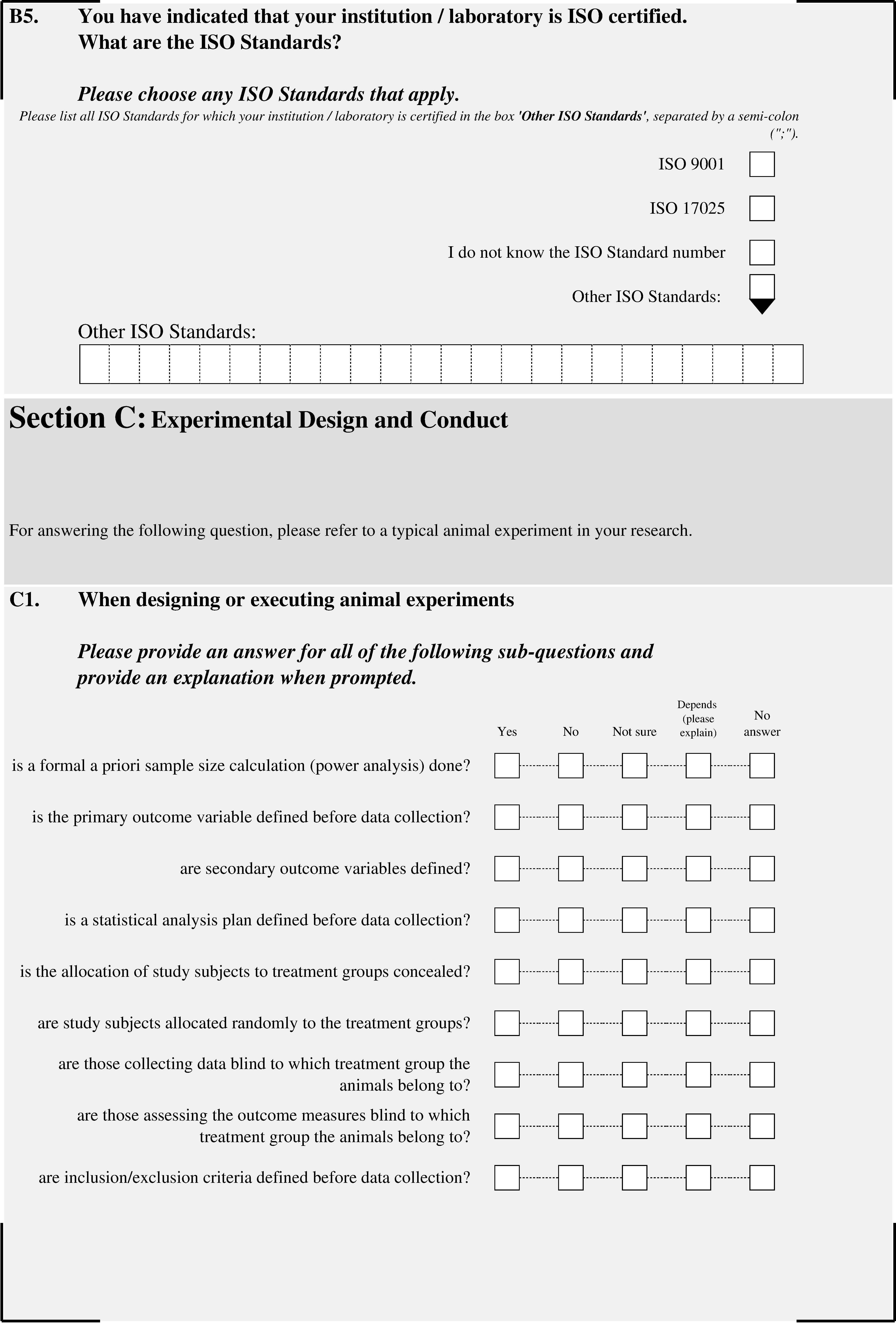
i


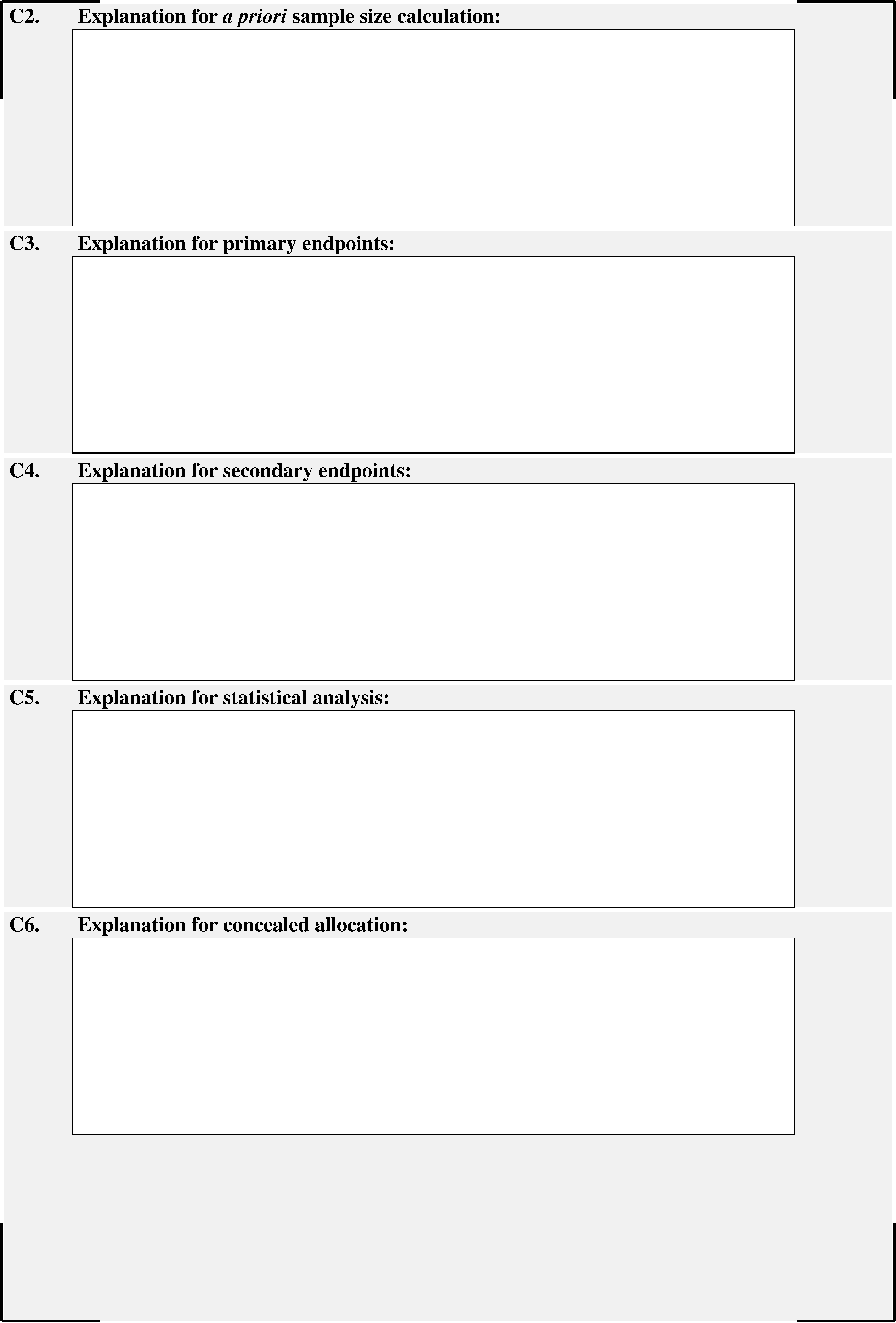


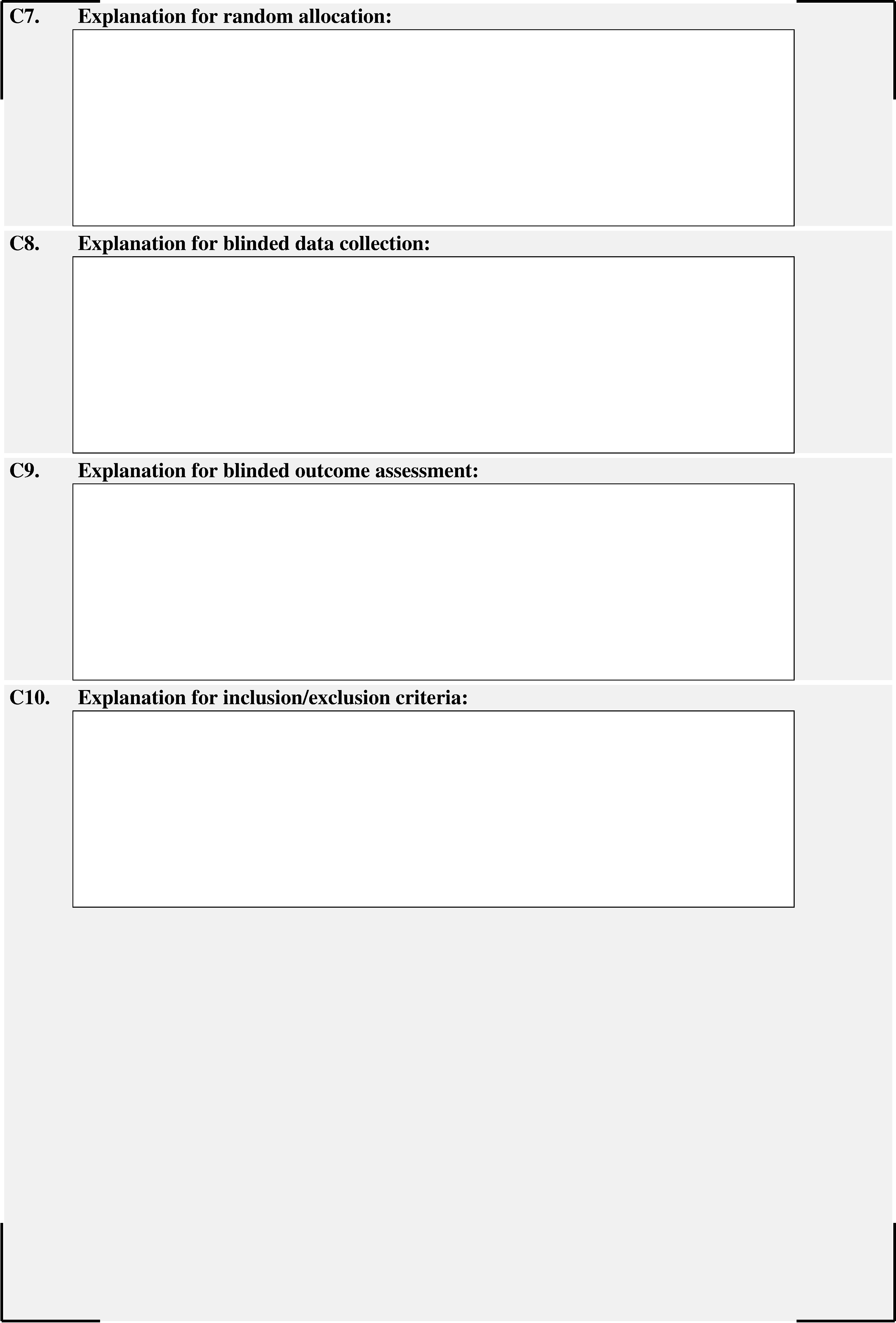


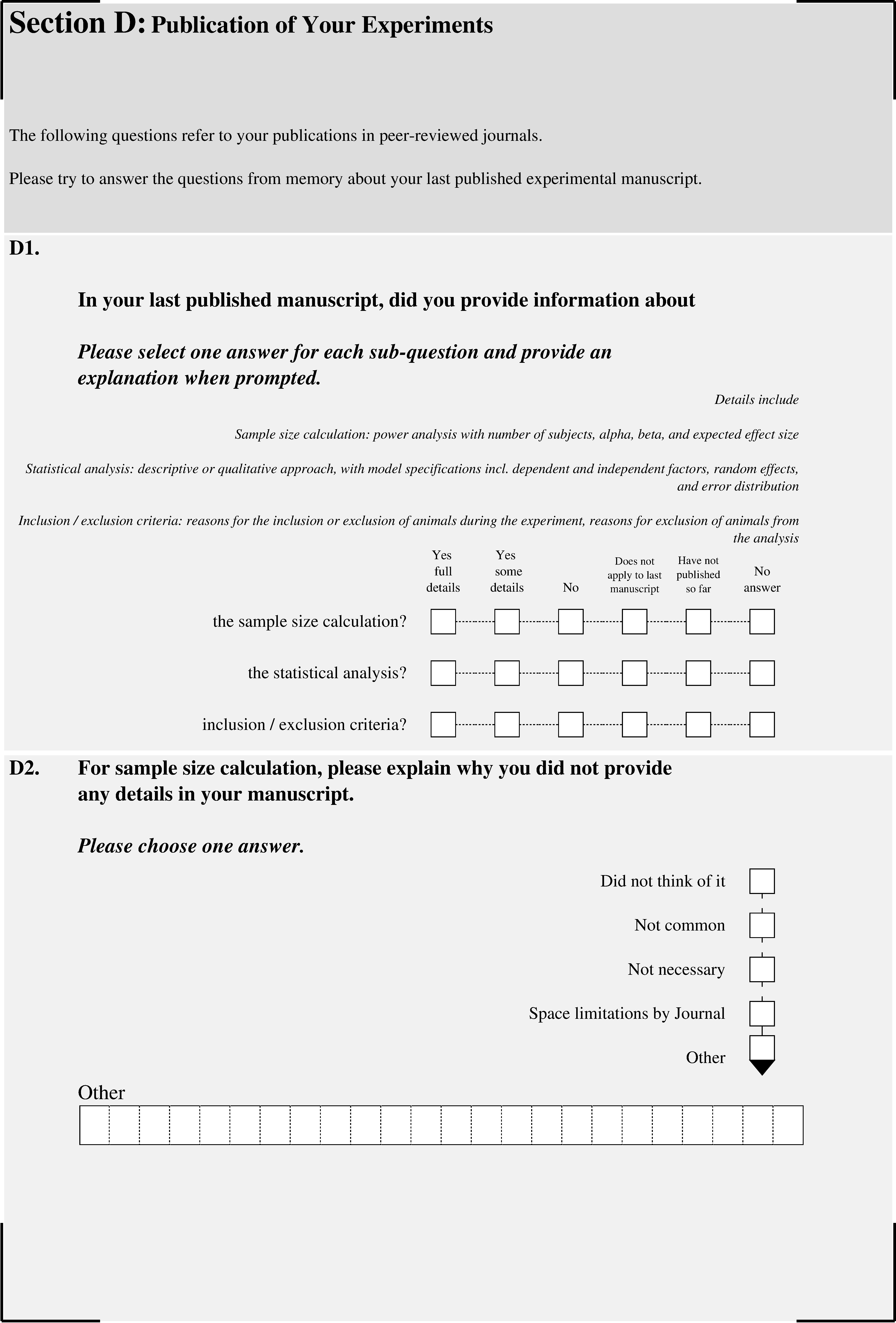
ii


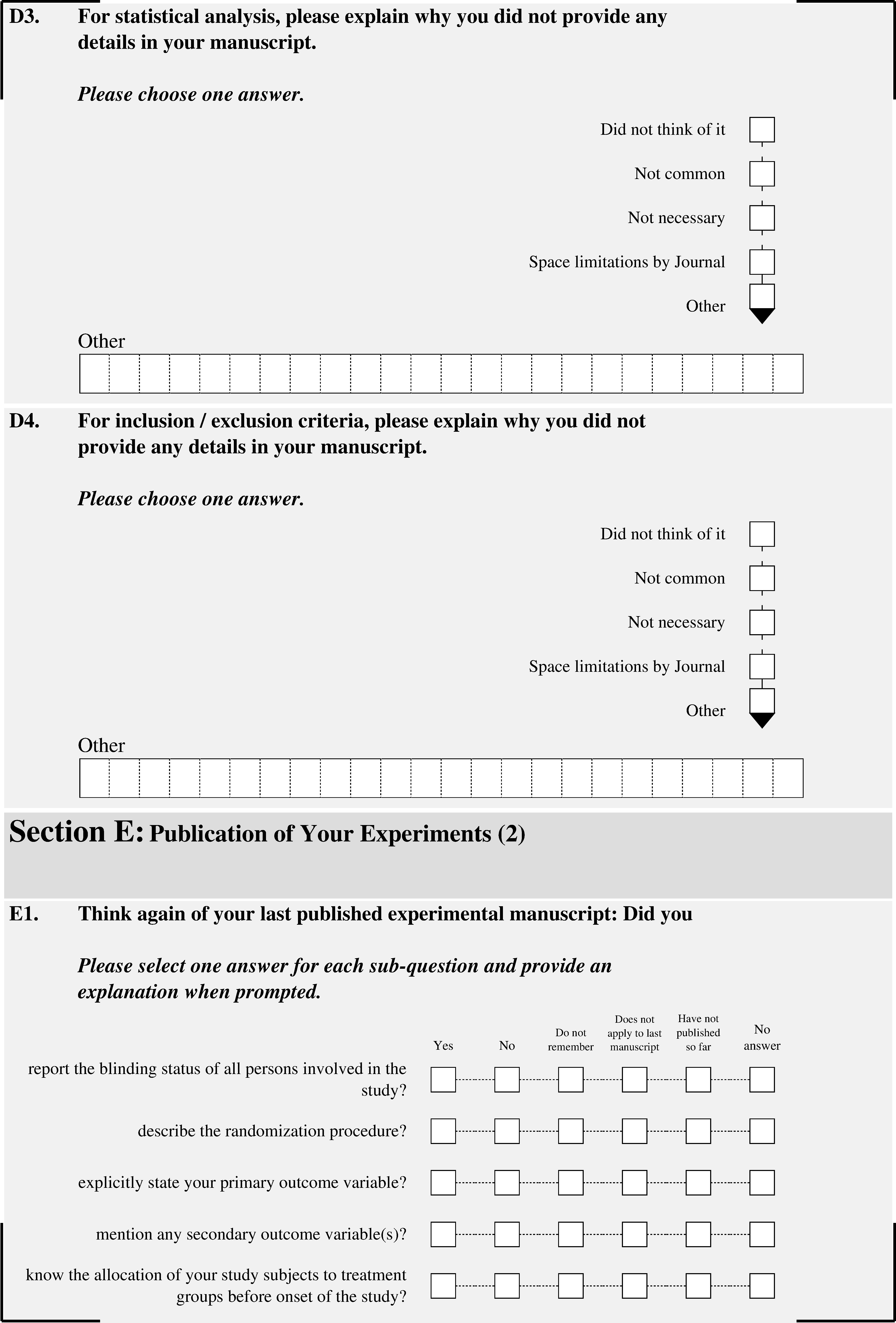
iii


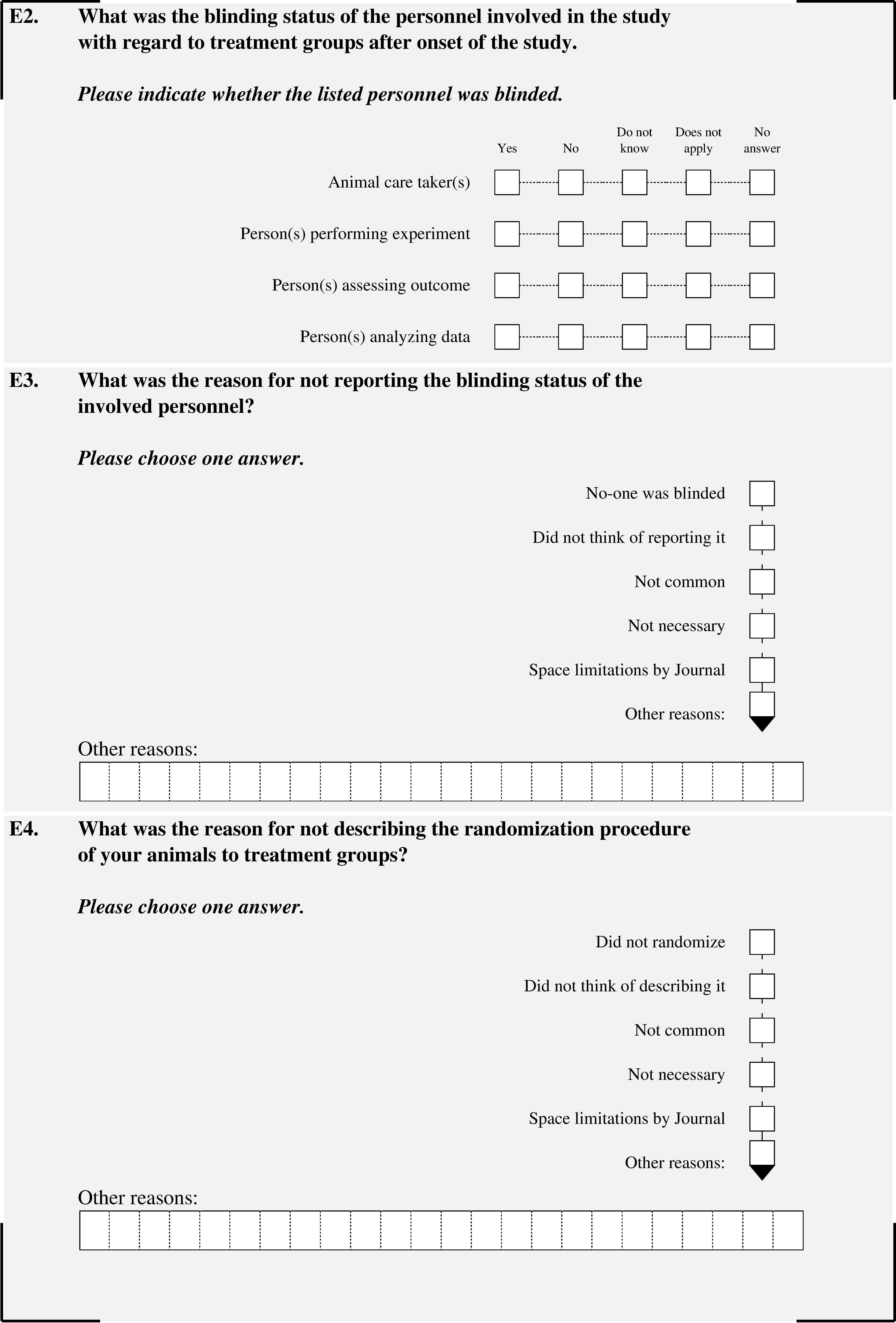


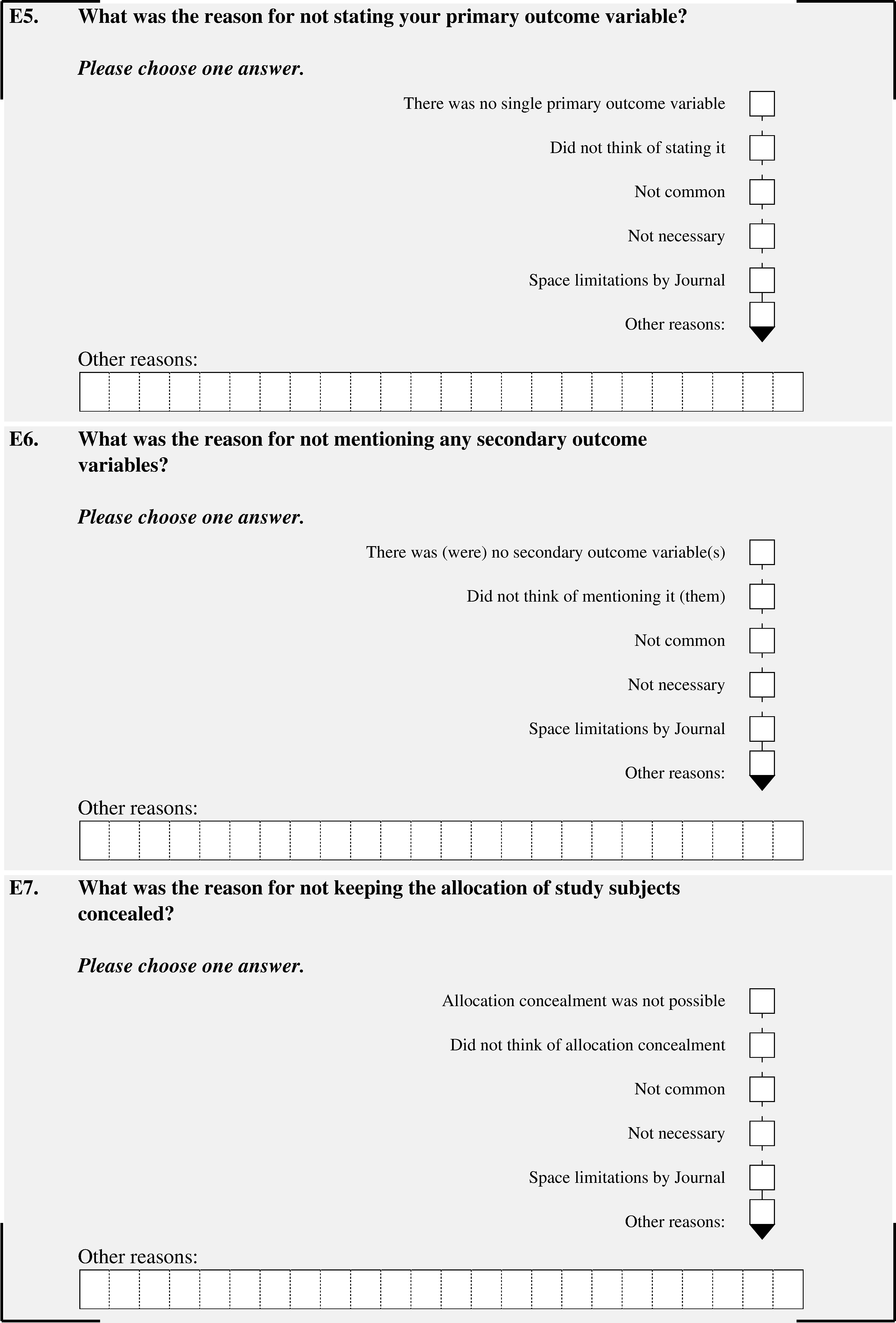


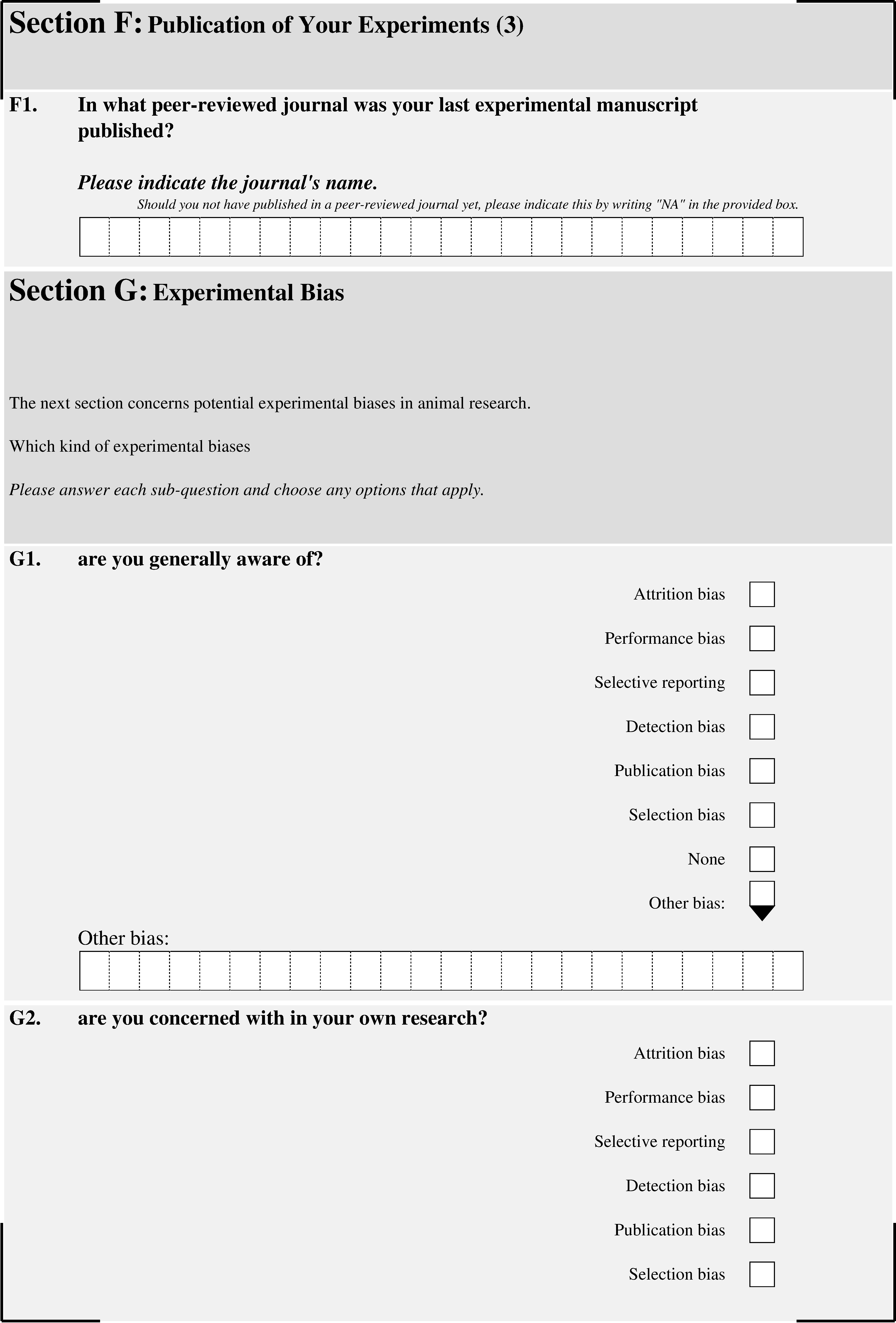


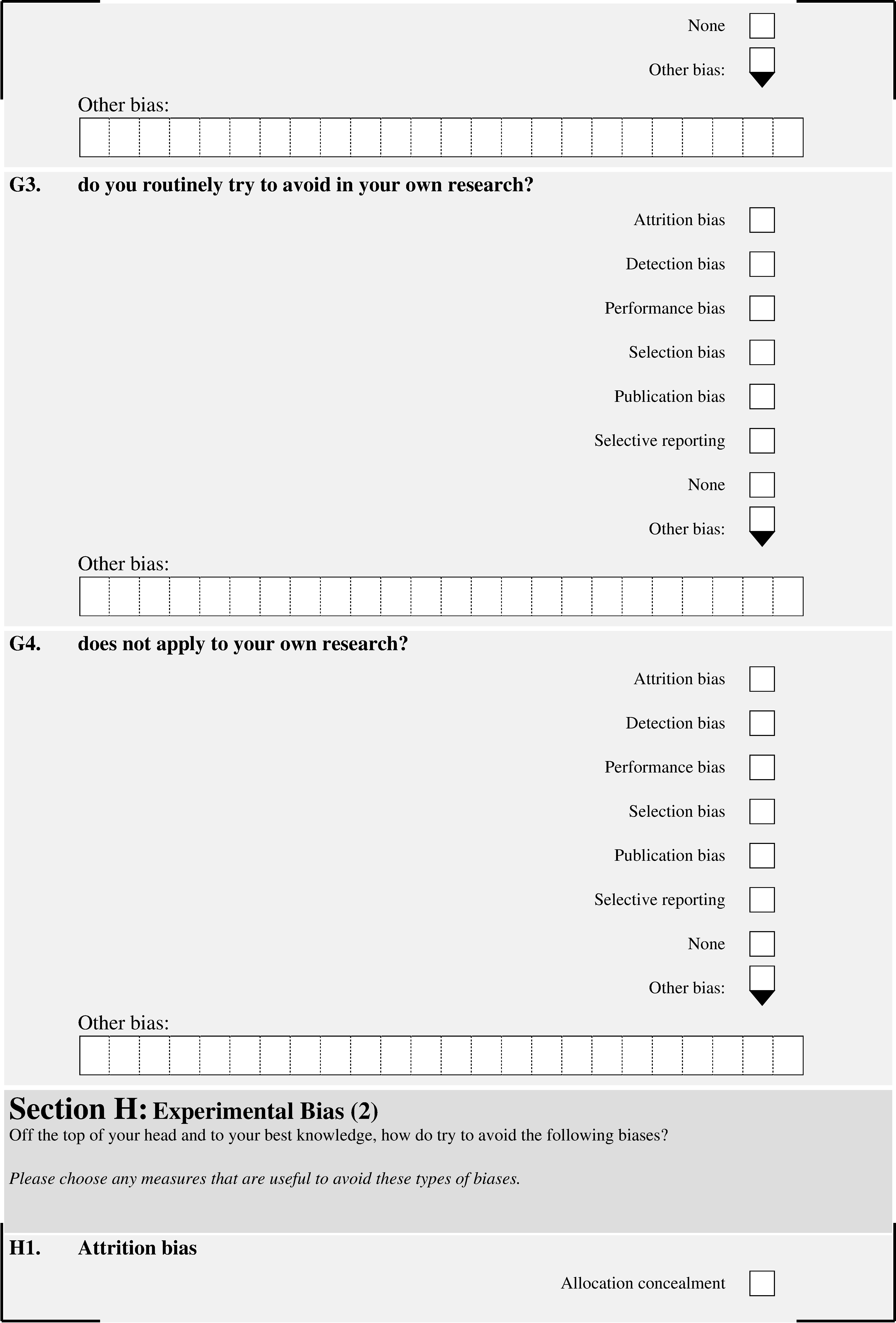


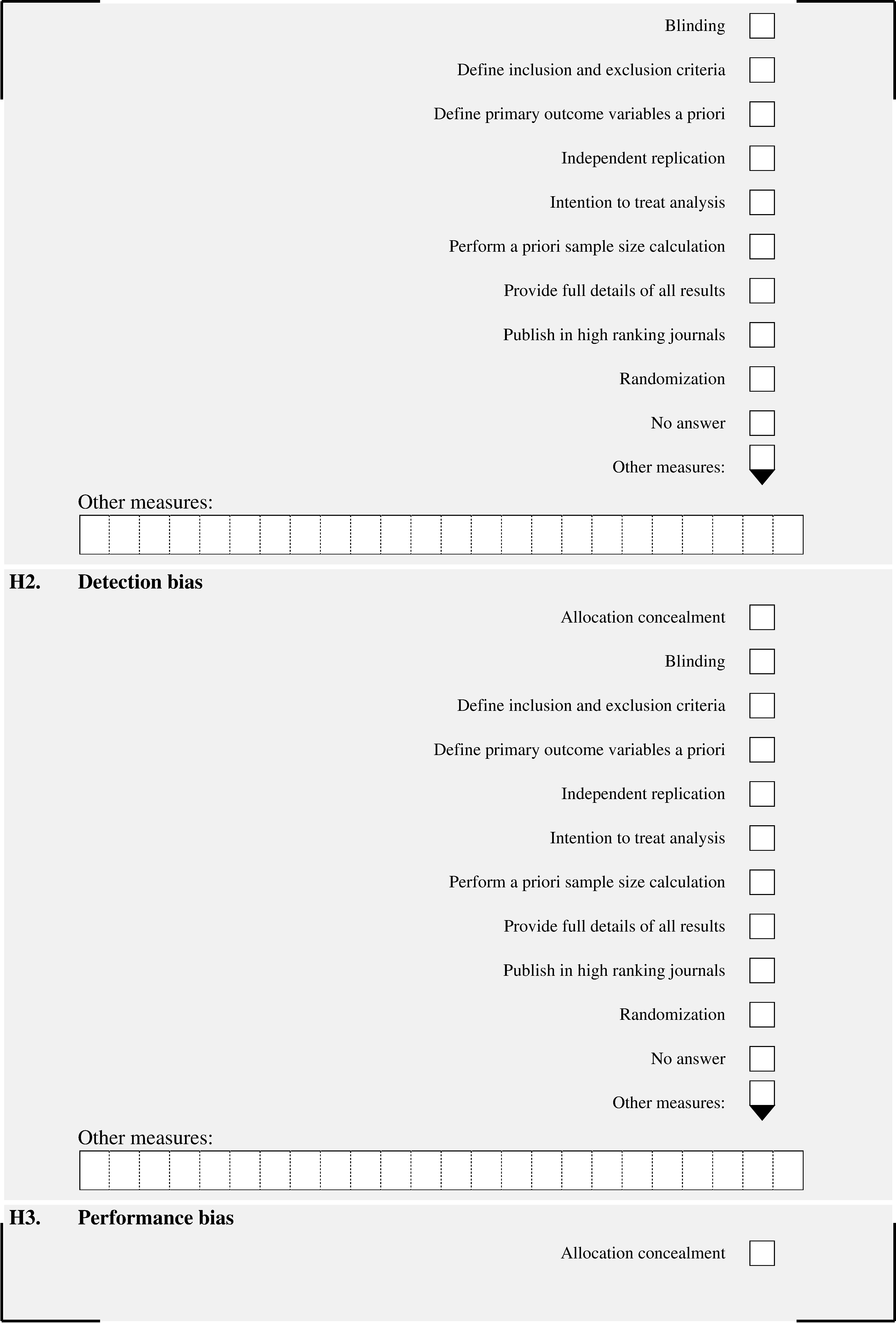


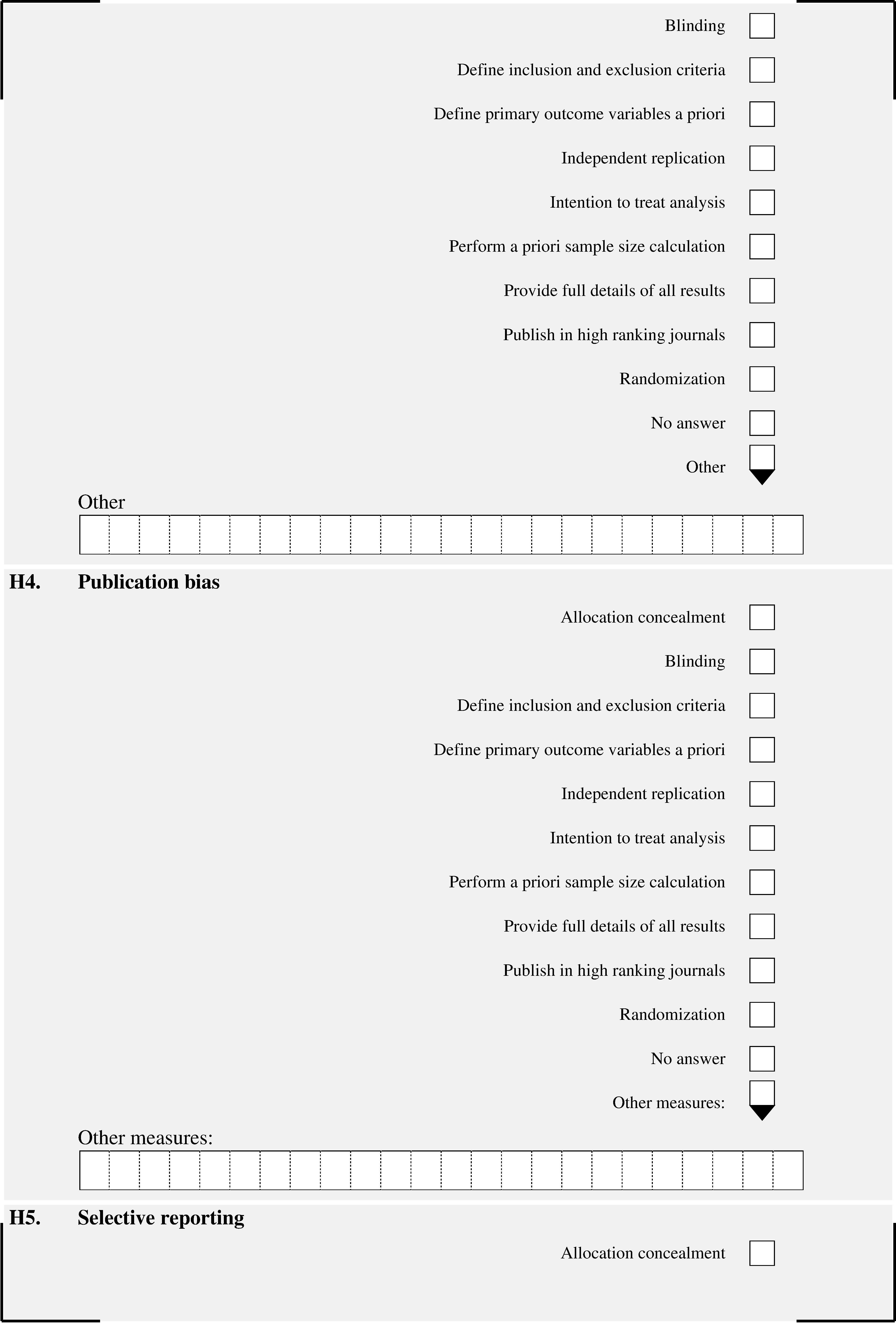


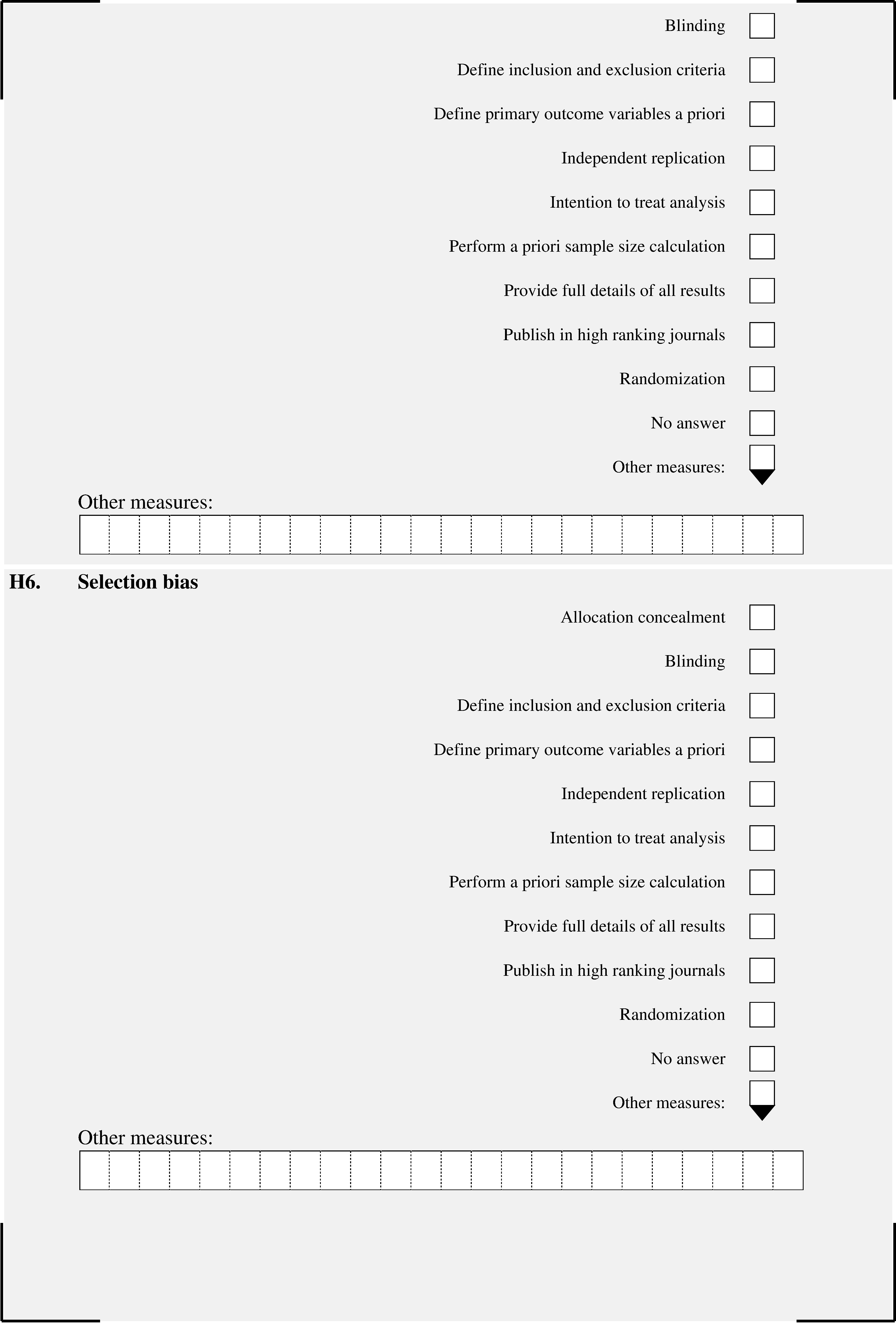


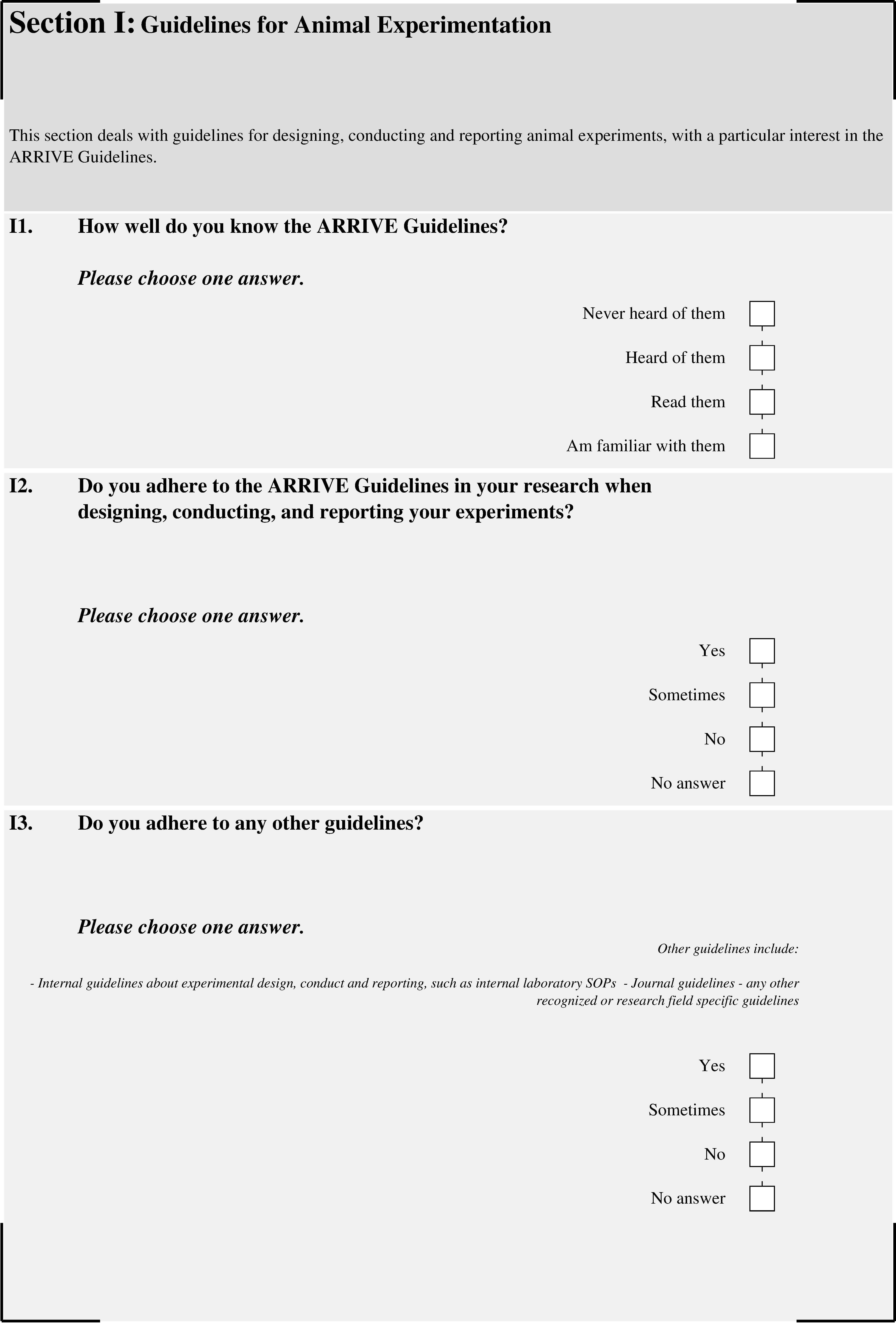


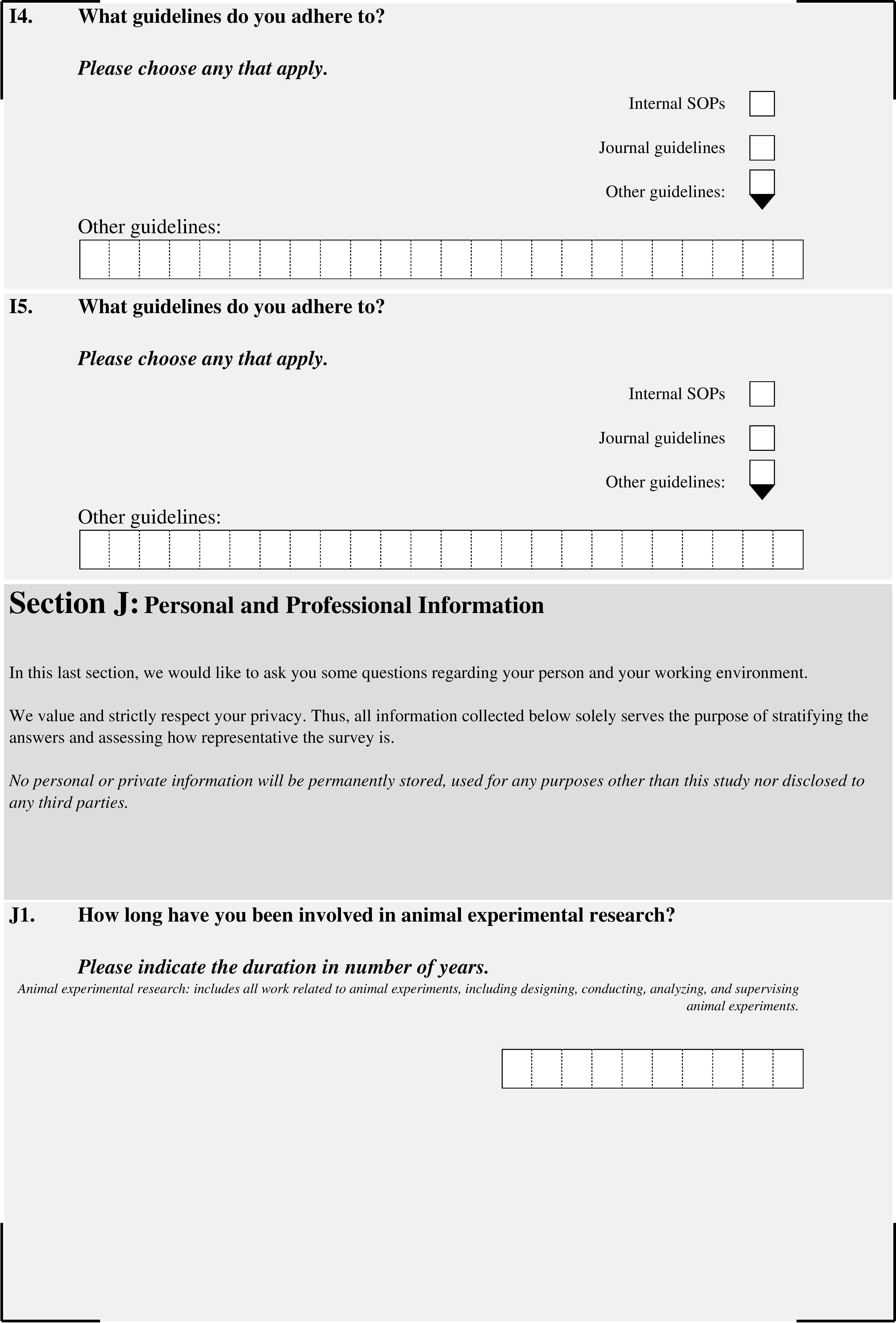


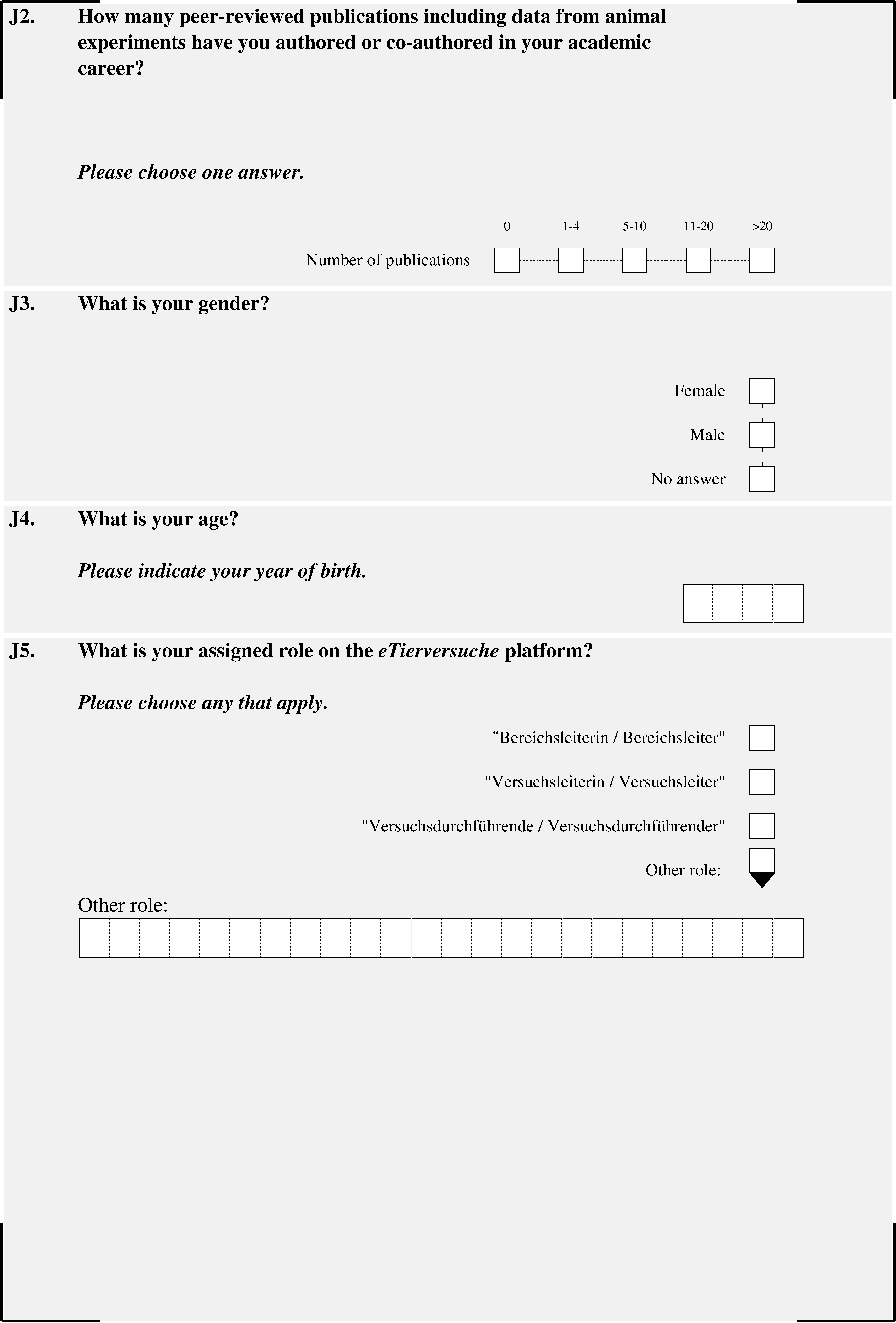


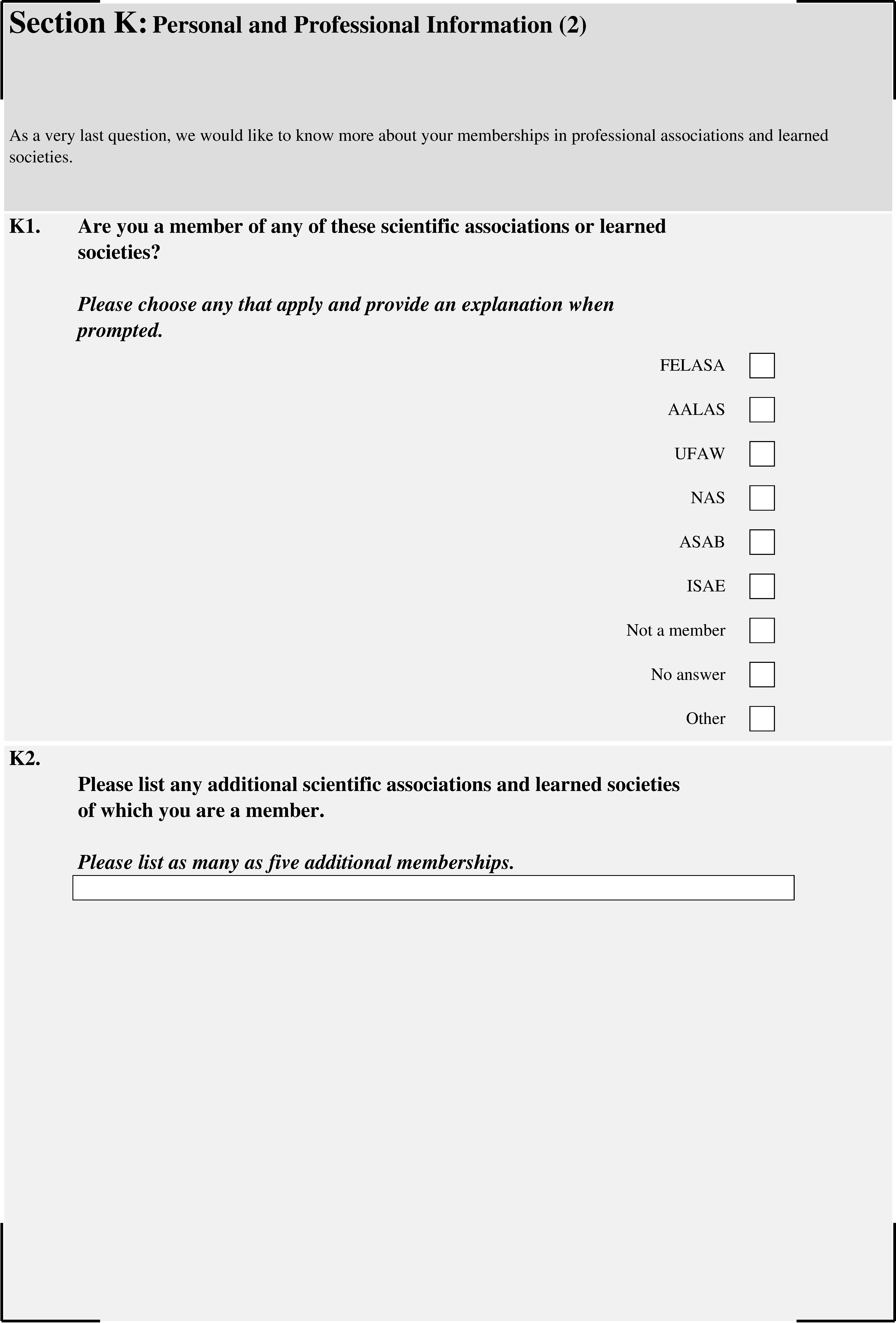

Supplement: S1 Text — (DOCX) [file pone.0165999.s004.docx]
