# Supplementary material for: The Researchers’ View of Scientific Rigor—Survey on the Conduct and Reporting of In Vivo Research
Source: PLoS One. 2016 Dec 2;11(12):e0165999. doi: 10.1371/journal.pone.0165999 (PMC5135049; doi:10.1371/journal.pone.0165999)
Supplement: S2 Text — (DOCX) [file pone.0165999.s005.docx]

**S2 Text. Personal Interviews**

In addition to the online survey, qualitative expert interviews, using the technique of a semi-structured guided interview [1], were conducted with a small sample of five participants of the online survey. Qualitative interviews are useful to assess subjective perceptions and opinions. To ensure high validity of such interviews, participants should be carefully selected to meet the purpose of the interviews (i.e. retrieving expert opinions) [2]. The five participants were selected to represent the broad range of animal research conducted in Switzerland, including ecology, molecular biology, veterinary medicine, pharmaceutical sciences, and neurosciences. They were sub-sampled from a pool of senior researchers included among the authors of a sample of 50 publications derived from a sample of 1277 licenses for animal experiments, which we had screened for prevalence of reporting measures against risks of bias as part of a related study reported elsewhere (Vogt et al, submitted).

The primary aim of the interviews was to obtain more detailed information on how exactly animal experiments were conducted, and how that relates to what researchers normally report in their publications, as well as assessing the range of measures used by researchers to avoid experimental biases. Furthermore, they were aimed to assess the researchers’ awareness of and personal opinion about the so called “Reproducibly Crisis” in animal research.

The interviews followed an interview guide, with a set of questions on the following topics: (i) awareness of the reproducibility crisis, (ii) reasons for this situation (incl. experimental biases), (iii) measures to ensure scientific validity of animal experiments, (iv) knowledge and use of reporting guidelines, (v) link between experimental conduct in the laboratory and reporting of experimental conduct in publications, (vi) personal information. Open questions were formulated allowing the interviewees to speak freely with regard to the different main questions. If necessary, the interviewer (TSR) asked prewritten follow-up questions, or cut question blocks short in case the answer was already provided during the conversation.

All interviews took place in the offices of the participants, were digitally recorded and lasted between 1.5 and 1.75 h. The complete interviews were transcribed for further analysis; interviewees were guaranteed complete anonymity.

**Interview Guide**

The interview followed a semi-structured interview. The interviewer (TSR) tried to pose all the main questions in the order of the interview guide. Depending on the course of the interview, some question blocks were cut short and some were expanded. The aim was to let the interviewees speak freely about their opinions, views and positions regarding the different issues, without interruptions. The following paragraphs are a copy of the interview questions in the order in which they were asked during the interview:

*i) Awareness of reproducibility crisis*

i.1) Does a problem with reproducibility exist in animal research, which is sometimes called by the media “Reproducibility Crisis”?

- Are you experiencing problems with reproducibility in your research field, or in Switzerland?

- How so? Please describe.

- How (strongly) affected is your scientific environment by this problem?

i.2) What is your opinion on the validity (internal and external) of animal experiments?

- How well suited are animal models (construct validity) which are used in animal research?

i.3) Do you see a link between poor reproducibility and poor scientific validity of animal experiments?

*ii) Reasons for situation*

ii.1) How did we end up in a situation as described before? Why is it a crisis?

- According to you, what could be the reasons for these problems?

- Could it be the high pressure of the scientific system resting on the scientists’ shoulders (e.g., publication pressure, performance pressure, financial pressure etc.)

- Or is it related to awareness / lack of knowledge about good scientific practice and how to design, conduct, and report science?

*iii) Measures to ensure scientific validity of animal experiments*

iii.1) Do you see any possible solutions to this problem, to the current situation? Which measures should be taken to find a way out of the crisis?

- What needs to be changed and where? Are there changes needed in the scientific system, in the way animal experiments are authorized, the way they are conducted, in the publication system, in education?

iii.2) What are the measures and precautions you are personally taking to guarantee high scientific validity and the highest possible quality of the research performed in your lab?

- How do you reckon do the various types of bias influence or affect the reproducibility crisis?

- Which biases are you concerned with in your own research, and which ones are you trying to avoid?

- How apt are the options for further education and how good is the education of doctoral students, postdocs etc. in your lab, in Switzerland, in general?

- Do you consult experts when designing an experiment or when analyzing your data?

- For example, are statisticians available who can help with statistical issues during data analysis? Are you taking advantage of such consulting services? Why not?

*iv) Knowledge and use of reporting guidelines*

iv.1) What do you think about guidelines on how to conduct and report animal experiments, which are becoming more and more common and recommended by various journals?

- Are these guidelines useful? What should be the purpose of such guidelines?

iv.2) From your point of view, how do guidelines help to increase or guarantee scientific validity of animal experiments? Do they help at all? Where could they help (e.g., for planning, executing or reporting animal experiments?)

- How important are the different items on such guidelines? How should they be weighted (e.g., the ARRIVE guidelines)?

- Which are the most important items on such checklists? (possible answers to choose from: blinding, randomization, allocation concealment, sample size calculation, inclusion/exclusion criteria, primary outcome variable, statistical analysis)

iv.3) Which guidelines do you know / are you familiar with, and are you using in your laboratory?

- Are these guidelines topic / research area specific?

iv.4) Which of the following guidelines do you know or have you heard of, read, are you applying?

□ ARRIVE Guidelines □ STAIR

□ CONSORT Statement □ Gold Standard Publication Checklist

□ REFLECT Statement □ other:

*v) Link between experimental conduct in the laboratory and reporting of experimental conduct in publications.*

v.1) How do you judge the quality, the diligence, and the adhering to scientific standards in the laboratory?

- What is your opinion about the claim that the low reporting rates of internal validity criteria in publications reflects the actual quality of experimental conduct in the laboratory?

v.2) Is there a “trust me” culture prevailing in animal experimental research?

v.3) Referring to this publication of yours, can you expand a bit on the design of this study?

- How was the study planned?

- How was the study conducted? (e.g., did you randomize, blind etc.)

- Were you involved in the design, conduct, and reporting of this study? What was your role?

v.4) Did you use or follow particular protocols or guidelines?

- What did these guidelines describe?

- How important was the adherence to good scientific practice standards in this study?

v.5) How did you assure (here, or in general) high scientific validity of this (other) studies?

v.6) When I am now confronting you with our evaluation of reporting internal validity criteria in the publication of this study, what was the reason for not reporting *these* validity criteria?

*vi) Personal information.*

vi.1) The last few questions are about your scientific career and about your person:

- How long have you been working in the field of animal experimental research?

- How many publications have you (co) authored in your career?

- Do you know your H-index, and what is it?

**Data Analysis**

Transcribed interviews were analyzed in 5 stages: formation of analytical categories, assembly of analytical guide, coding according to guide, quantification of interview material, and finally case interpretation [3].

**Results**

The qualitative analysis of the interviews provided in depth personal opinions by the five interview partners on relevant aspects related to scientific rigor, risks of bias, and the “Reproducibility Crisis” in general, and were used to facilitate interpretation of the quantitative results of the online survey. It was not intended to provide a full analysis of all questions, but rather highlight relevant statements of the participants. This was either done in the discussion of the main article directly with quotation marks or as an interpretation of answers; no reference to the interviewees was made, however, in order to guarantee their anonymity.

The results of the five interviews indicated that researchers commonly use more measures to avoid risks of bias than that they report in their published work. Reasons mentioned by the interviewees why they did not report such measures against bias were that “the journal did not require to describe it [e.g., randomization]”, “certain things are self-evident and do not need to be reported”, and it is considered “good scientific practice” to keeping those standards, thus they need not be described. One interviewee justified the non-reporting of criteria of good research practice by stating that “There is a threshold for what is relevant to the own laboratory and to the research community outside the laboratory”. Also, space limitations were still an issue and a reason for not reporting certain information about the design and conduct of a study.

A quantitative approach was used to compare an example of the participants’ publications and the interviewees’ answers about which measures against risks of bias they were typically using in their research. According to this comparison there were at least six cases of specific measure to avoid risks of bias that were used during experimental conduct but not reported in the published manuscript. In general, less than half of the measures against risks of bias studied here (10 of 24 applicable measures) were actually used by the researchers, and even less were reported (5 of 33 applicable measures). Confronted with the low reporting of measures against risks of bias in their publications, interviewees mentioned that certain criteria were not required by journals, some criteria were not applicable, or they did not know how to perform a sample size calculation for that particular study design. Space limitations were explicitly mentioned, as well as not being aware of the importance of these measures to avoid experimental bias.

According to the interviews, a general “trust-me culture” would exist in the life and biological sciences towards excellent research being performed [see also 4]. Mistrust would do more harm than good to the reproducibility crisis, and checklists about scientific standards prior to publication would not increase the researchers’ trust in the scientific system.

**References**

1. Flick U. An introduction to qualitative research. 4th ed. Flick, editor. London, Thousand Oaks, New Delhi: SAGE Publications Ltd; 2009.

2. Creswell JW. Collecting data in mixed methods research. In: Creswell JW, Plano Clark VL, editors. Des. Conduct. Mix. Methods Res. 2nd ed. Thousand Oaks, CA: SAGE Publications, Inc; 2006. p. 110–27.

3. Schmidt C. The analysis of semi-structured interviews. In: Flick U, von Kardorff E, Steinke I, editors. A companion to Qual. Res. [Internet]. 1st ed. London, Thousand Oaks, New Delhi: Sage Publications; 2004. p. 253–8. Available from: sdhprc.ir/download/A_Companion_to_qualitative_research.pdf

4. Siebert S, Machesky LM, Insall RH. Overflow in science and its implications for trust. Elife [Internet]. 2015;4:1–7. Available from: http://elifesciences.org/lookup/doi/10.7554/eLife.10825
